# Long-term functional rescue of trauma-induced vision loss by a novel, small molecule TrkB modulator

**DOI:** 10.1371/journal.pone.0320231

**Published:** 2025-09-29

**Authors:** Shweta Modgil, Christopher L. Walker, Micah A. Chrenek, Hans E. Grossniklaus, Frank E. McDonald, P. Michael Iuvone

**Affiliations:** 1 Department of Ophthalmology, Emory University School of Medicine, Atlanta, Georgia, United States of America; 2 Department of Chemistry, Emory University, Atlanta, Georgia, United States of America; Transilvania University of Brasov: Universitatea Transilvania din Brasov, ROMANIA

## Abstract

Brain-derived neurotrophic factor (BDNF) signaling through the tropomyosin-related kinase B (TrkB) receptor promotes neuronal growth and survival following an injury. However, its short half-life and pleiotropic effects limit the clinical use of BDNF as a therapy in neurodegenerative disorders. Identification of novel and selective TrkB activators may ameliorate the damage caused to retinal neurons during eye-related injuries and may reduce adverse visual outcomes associated with visual trauma. We previously described a small molecule, N-[2-(5-hydroxy-1H-indol-3-yl) ethyl]-2-oxopiperidine-3-carboxamide (HIOC), that activates TrkB and reduces the decline in visual function in a mouse model of ocular trauma. Using the lead optimization approach, we subsequently synthesized a fluoropyridine analog of HIOC, 2-fluoro-N-(2-(5-hydroxy-1H-indol-3-yl) ethyl) nicotinamide (HIFN), which also successfully activates TrkB. HIFN is a more potent TrkB modulator than the parent compound, HIOC. Further, treatment with HIFN demonstrated neuroprotection in an animal model of overpressure ocular blast injury, ameliorating blast-related visual functional decline. Mice treated with HIFN had better visual acuity, contrast sensitivity, and retinal function supported by enhanced survival of retinal ganglion cells compared to vehicle-treated animals. Moreover, HIFN exhibited better protective effects than HIOC. The therapeutic effects of HIFN were attributed to TrkB activation, as blocking the receptor with a selective receptor antagonist (ANA-12) abrogated the neuroprotection. Together, our results identify HIFN, a novel TrkB receptor modulator, as a strategy for decreasing retinal degeneration and progressive vision loss associated with traumatic ocular injury. In addition, this compound may have broader applications in treating other diseases with altered TrkB activity.

## Introduction

BDNF is an endogenous ligand of the TrkB receptor [1], which, upon its binding, induces receptor dimerization and autophosphorylation of tyrosine residues in the intracellular kinase domain of the receptor [2]. TrkB/BDNF through downstream signaling molecules, phosphatidylinositol 3-kinase (PI3K)/Akt, mitogen-activated protein kinase (MAPK)/Erk, or phospholipase (PLC-γ), regulates the normal functioning of neurons and promotes survival following damage to neurons [3]. TrkB/BDNF signaling has important roles in a wide range of neurodegenerative disorders ranging, from Alzheimer’s and Parkinson’s diseases [4], amyotrophic lateral sclerosis, to optic neuropathies [5], as well as psychiatric disorders such as depression [6].

TrkB activation is protective in traumatic brain injury (TBI). TBI is common among civilians in blast-prone areas and is suffered by many military personnel in war zones [7]. According to a report by Health.mil, 515,885 United States service members sustained at least one blast injury between 2000 and 2024 Q2 (https://health.mil/Military-Health-Topics/Centers-of-Excellence/Traumatic-Brain-Injury-Center-of-Excellence/DOD-TBI-Worldwide-Numbers). With better-designed protective gear, the mortality in such cases has decreased, but the morbidity associated with these blasts is much more prevalent. Fall-related injuries in the elderly, automobile accidents, and sports injuries are also significant causes of traumatic injuries. These injuries are often accompanied by visual impairment; 75% of TBI patients are reported to have vision-related problems [8]. Traumatic injury can include rupture of the eye globe or penetration by foreign objects, resulting in an open eye injury. However, in closed globe injuries, the corneoscleral junction often remains intact, but superficial or intraocular injury is present. These injuries are more difficult to address because they usually go unnoticed, manifesting slowly without any visible symptoms. By the time they are detected, vision loss is often irreversible. Trauma-induced visual deficits may arise due to loss of retinal ganglion cells (RGCs) and optic nerve degeneration that disrupts the signal transduction to higher brain centers. At present, there are no effective treatments for such traumatic optic neuropathy. While TrkB activation is protective in TBI [9–11], few studies focus on vision.

**Fig 1:**
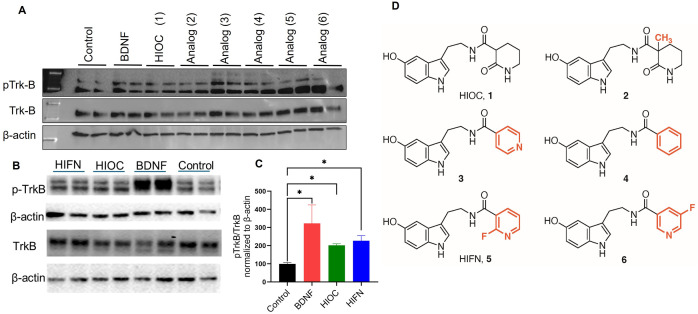
TrkB activation by HIOC and analogs: HIFN *vs* HIOC. (A) Cell-based screening of HIOC analogs in primary cortical neurons. Analogs were tested at 10 nM concentration, and TrkB phosphorylation state was assessed using western blot; BDNF was tested at 4 ng/ml. Analogs **5** (HIFN) and **6** both activated TrkB and were better than HIOC. (B) TrkB activation by HIFN was also confirmed by pTrkB expression in NIH-3T3 cells expressing human TrkB after stimulation with analogs (10 nM) or 8 ng/ml of BDNF. Immunoblots are presented in S1 raw_images.pdf. (C) Quantitative analysis of TrkB phosphorylation; levels of phosphorylated TrkB were normalized to total TrkB and expressed as a percentage of control (control cells were treated with DMSO, which was used to dissolve analogs). ^*^ p ≤ 0.05 vs Control; n = 5. (D) Chemical structure of parent molecule HIOC (**1**) and analogs of HIOC tested *in vitro*.

Although therapeutic effects of BDNF are evident from preclinical studies, clinical utility is limited by the short biological half-life of BDNF and its inability to cross the blood-brain barrier (BBB) and blood-retina barrier (BRB) [12–13]. This has steered the field to develop alternative molecules that specifically activate the TrkB receptor in the central nervous system upon systemic administration. In this direction, synthetic peptide mimetics of BDNF, including essential sequences for TrkB interaction stimulated TrkB [14] but were susceptible to proteolytic degradation. To overcome this challenge, in the last decade research has shifted towards finding potent small molecule TrkB agonists with the desired properties of penetration to the central nervous system from the systemic circulation and more stability than BDNF. We previously reported a selective TrkB modulator, HIOC (**1**, Fig 1), which passes the BRB and BBB, has a relatively long half-life *in vivo*, and mitigates visual function decline [1]. In the present study, using HIOC as a lead compound, we synthesized various analogs and tested them for TrkB activation, as measured by phosphorylation state. Our data identifies HIFN (**5,** Fig 1), an analog of HIOC, as a novel and potent TrkB modulator, and provides proof of concept for its efficacy in reducing ocular trauma-induced visual dysfunction.

## Materials and methods

### Analog synthesis

The synthesis of HIOC analogs generally followed the *N*-acylation protocol developed for the synthesis of HIOC (**1**) [15], from serotonin hydrochloride and the corresponding carboxylic acid (S1-S6 Figs in S1 File). These optimized conditions favored selective acylation of the primary amine of serotonin hydrochloride via *N*-acylimidazole intermediates.

### Primary culture and NIH-3T3-TrkB cell line culture

Timed pregnant Sprague Dawley rats were procured from Charles River Laboratories. Primary cortical cultures from embryonic day 18 rats were prepared as previously described by Pacifici and Peruzzi [16].

NIH-3T3 cells expressing human TrkB (NIH-3T3-TrkB) were provided by Frank Longo, Stanford University, and were propagated in DMEM medium (ATCC-30–2002) supplemented with 10% calf serum (ATCC-30–2030), penicillin/streptomycin (100 I.U./mL penicillin and 100 μg/mL streptomycin, P4333, Sigma-Aldrich, St Louis, MO). For protein recovery experiments, 3*10^5^ cells were plated on 6-well poly-D-lysine-coated plates (Corning) and protein extraction was carried out using cell lysis buffer (Invitrogen #FNN0011, Carlsbad, CA) containing protease inhibitor (Roche, #11836153001, Basil, Switzerland) and phosphatase inhibitor (Roche, #04906845001).

### Stimulation with analogs

Cells were serum deprived overnight to avoid the possible exposure of cells to BDNF present in serum and were stimulated with various analogs at 10 nM concentration. BDNF (#SRP3014−10UG, Sigma) was used as a positive control for TrkB phosphorylation. Thirty minutes after the addition of test compounds, cells were washed with ice-cold PBS and treated with ice-cold lysis buffer prepared as described above. Cell lysate was collected and centrifuged at 10,000 rpm for 10 minutes @ 4°C and stored at –20°C until further use.

### Western blotting

Total protein estimation in cell lysates was done using BCA (#23227, Thermo-Scientific). Protein lysate (50 µg) was prepared for each sample using a 4X Laemelli buffer (#1610747, Bio-Rad). Proteins were separated on 7.5−15% gradient polyacrylamide gels (#5671085, Bio-Rad) using tris-glycine-SDS buffer (#1610732, Bio-Rad) followed by transfer to 0.45 mm PVDF membranes (Midi PVDF Transfer Packs, #1704157, Bio-Rad) for antibody staining. The membrane was blocked in 5% bovine serum albumin (BSA) (#37520, Thermo-Scientific) in Tris-buffered saline-0.1% Tween 20 (TBST) for 30 minutes at room temperature. Primary antibody incubation was carried out [anti-phospho TrkB (#4619, Cell Signaling), anti-TrkB (#92991, Cell Signaling), and anti-β-actin (#4970, Cell Signaling)] overnight at 4°C, followed by washing the next day. Blots were then incubated with horse radish peroxidase (HRP)-conjugated secondary antibody (#7074S, Cell Signaling) for 1 h at room temperature, developed with HRP substrate (#WBLUR0500, Millipore), and imaged using a ChemiDoc Imaging System (Bio-Rad).

### Animal and ethics statement

C57BL/6J mice, 2–3 months old (N = 305; 257 males and 48 females), were procured from Jackson laboratories (Bar Harbor, ME, USA). All animal experiments were conducted in accordance with guidelines of the National Institutes of Health Care and Use of Laboratory Animals and were approved by Emory University’s Institutional Animal Care and Use Committee (PROTO201700188) and the US Army Animal Care and Use Review Office (VR170139.e001). Mice were maintained in a standard 12:12 h light/dark cycle with free access to food and water and monitored daily. Mice were euthanized by asphyxiation with carbon dioxide from a bottled source, followed by cervical dislocation. Humane endpoints, as specified by Policy 357 of the Emory University Institutional Animal Care and Use Committee, were applied as necessary prior to the planned experimental endpoint.

### Experimental design

#### Ocular blast.

Animals were subjected to a blast of air directed at the front of the right eye, as described previously [17]. Briefly, the mouse was anesthetized by intraperitoneal (ip) injection of a mixture of xylazine (10 mg/kg) and ketamine (100 mg/kg) and received acetaminophen (4% solution) orally. It was positioned in a plexiglass sleigh that slides into a plastic polyvinyl chloride cylinder. This hollow cylinder had a hole equal to the diameter of the muzzle of the air gun. The position of the mouse was adjusted in a way that the right eye of the mouse was in line with the hole; the head was supported with padding to prevent secondary injuries. The cylinder provides protection to the rest of the body except the eye. The pressure was calibrated with a pressure transducer (Honeywell SensorTec sensor, Model STJE, 0–100 psi) at the muzzle of the gun, and a blast of compressed air (~20 psi at the level of the eye) was delivered. After induction of blast, the eye was kept moist with Optixcare eye gel (Aventix, Irvine, CA) to prevent corneal drying and desiccation. Animals were placed on a heating pad and allowed to recover from anesthesia, aided by administration of atipamezole (0.5 mg/kg ip). Sham animals were treated identically except for exposure to air pressure waves. A total of 233 mice were subjected to the blast or sham protocol; 14 mice died subsequent to blast and prior to the experimental endpoint.

#### Toxicity testing.

For acute toxicity testing, groups of male (N = 6) and female (N = 6) mice were injected ip with HIFN (300 mg/kg or 600 mg/kg) or with vehicle. The mice were monitored for signs of toxicity, including change in fur color, lethargy, red secretions around the eye, hunched back, tremors, and mortality, hourly for the first 6 hours and daily for the next 6 days. For chronic toxicity testing, groups of male and female mice received daily ip injections of 40 mg/kg of HIFN or vehicle for 40 days. Mice were euthanized approximately 24 hours after the last injection; blood and serum samples were collected for complete blood count and serum biochemistry analyses by the Emory University Department of Veterinary Services. Brain, liver, kidney, spleen, and heart were dissected and fixed in 10% formalin and sent to the L.F. Montgomery Pathology Laboratory of the Emory Eye Center; fixed tissues were sectioned at 7 μm thickness, stained with hematoxylin and eosin and examined by light microscopy at 20X magnification by the laboratory’s pathologist (HEG). A total of 72 mice were used in this protocol; no mice died prior to the experimental endpoint.

### Drug preparation and treatment

HIOC (**1**) was synthesized as described in Setterholm et al. [15]. HIFN (analog **5**) and other analogs were synthesized as described in Supplemental Information (S1 Fig in S1 File). Stock solutions of HIOC and HIFN were prepared in 100% dimethyl sulfoxide (DMSO) and further diluted with the final solution containing 10% DMSO, 16.5% Cremophor EL (Sigma-Aldrich St. Louis, MO), and 16.5% ethanol in PBS. Stock solutions of ANA-12 (Sigma-Aldrich St. Louis, MO) were prepared using the same formulation as for analogs. For *in vitro* experiments, 10 nM of analogs were added to the culture medium. For animal studies, unless noted otherwise, mice were injected intraperitoneally (ip) with either analog (40 mg/kg) or vehicle, 30 min after blast and then daily at approximately the same time of day for the next 6 days (7 doses in total). For the ANA-12 experiments, the mice were pretreated (0.5 mg/kg, ip) 2.5 h prior to the daily injection of HIFN.

### Contrast sensitivity and visual acuity testing

Visual functions in animals were measured using optomotry (Cerebral Mechanics. Inc., Lethbridge, Alberta, Canada). Contrast sensitivity was measured at a spatial frequency of 0.064 cycles/degree, which is the optimal spatial frequency for C57BL/6J mice [18]. Briefly, animals were kept on a raised platform inside a closed chamber surrounded by LED display screens that generate a virtual rotating drum of vertical, sinusoidal light and dark bars. The chamber was mounted with a video camera to observe the animal’s behavior in real time. Alternating light and dark vertical stripes moving in either clockwise or anticlockwise direction were presented to the mouse, triggering an optomotor reflex. The contrast sensitivity threshold is the lowest contrast at which mice showed the visual reflex, and data are presented as the inverse of the contrast threshold. To evaluate visual acuity, the animal was shown alternating black and white gratings (at fixed contrast of 100%) of increasing spatial frequency, starting with a frequency of 0.039 c/d to find the animal’s spatial frequency threshold. Contrast sensitivity follows a circadian rhythm [19]; therefore, all recordings were made between 11am to 3 pm. The data are presented as a percentage of the sham control for contrast sensitivity and visual acuity.

### Scotopic electroretinography (ERG) and pattern ERG (PERG) recordings

Animals were dark-adapted overnight and prepared for ERG recordings under dim red light (<3 lux). Mice were anesthetized and pupils dilated with topical application of 1% tropicamide ophthalmic solution (Akorn, Inc., Lake Forest, IL), followed by application of 1–2 drops of proparacaine hydrochloride (0.5% ophthalmic solution, Akorn, Inc.). Body temperature was maintained with a heated platform integral to the Celeris ERG system (Diagnosys, Lowel, MA). A layer of Optixcare gel was applied to the cornea, and stimulators were positioned with the gel, making a thin layer between the cornea and stimulators. For scotopic ERG measurements, simultaneous recording of both eyes was carried out using an intensity series of white flashes ranging from 0.01 to 10 cd*s/m^2^.

PERG was recorded at 100% contrast. A stimulus of horizontal gratings with contrast reversal was used with the following specification: spatial frequency of 0.155 c/d, 2.1 contrast reversal per sec and mean luminance of 50 cd/m^2^. In total, 600 sweeps were averaged to get the final measurements. The PERG waveform is characterized by a small negative wave with an implicit time of approximately 50 msec (N1), a second wave in the form of positive deflection, peaking at approximately 80 msec (P1), followed by a late negative peak at around 350 msec (N2). PERG P1 and N2 wave amplitude ± standard error of the mean (SEM) are plotted for all groups to determine the functional activity of RGCs.

### Retinal whole-mount staining

Animals were sacrificed, eyes were enucleated and fixed in 4% paraformaldehyde for 1 hour. The eye was dissected along the limbus, and the anterior portion containing the lens and vitreous was discarded. The eyecup with retina was flattened by making radial cuts along the nasal-temporal and inferior-superior retina. The retinal whole-mount was washed with 1X PBS to remove any attached vitreous or iris and blocked with blocking buffer (10% donkey serum, 5% BSA, 0.5% tritonX-100 in PBS) for 1 hour at room temperature (RT). Overnight incubation at 4°C in primary antibody [anti-Brn3a; # sc-8429, Santa Cruz or anti-RNA Binding Protein With Multiple Splicing (RBPMS) # ABN1376, Millipore Sigma] diluted in 0.5% Triton X-100/PBS (1:500) was done. The next day, following washing in 1X PBS-0.1% Tween20 (3 times), retinas were incubated in secondary antibody diluted in 1X PBS (1:1000) for 2 hours at room temperature (RT) in the dark. The secondary antibody was removed, and retina was washed with PBS three times (10–15 min each, in the dark). The retina was finally transferred to a glass slide and mounted in Vectashield mounting medium (Vector Laboratories, Newark, CA). Imaging was done with a Nikon Ti2 microscope with a Nikon A1R confocal imager. RGCs were counted in Image J using the RGC plug-in.

#### Statistics.

Data are expressed as mean ± standard error of the mean (SEM) and were analyzed by Student’s t-test for comparisons between two groups. For multiple comparisons, one- or two-way analysis of variance as appropriate, with Tukey’s post hoc test was applied using GraphPad Prism 9. If the data failed the equal variance test, they were analyzed using the Kruskal-Wallis test.

## Results

### HIFN, a novel TrkB modulator

The synthetic methods and characterization of the HIOC analogs studied are provided in Supporting Information (S1-S6 Figs in S1 File). We conducted cell-based screening of HIOC analogs to evaluate their efficacy in activating TrkB receptor, by employing two different cell systems (Fig 1). A fibroblast cell line (NIH-3T3-cells) stably expressing human TrkB receptor and primary cortical neurons were stimulated with various analogs for 30 minutes. Compounds **5** and **6** exhibited TrkB phosphorylation similar to BDNF, utilized as a positive control. Notably, both compounds demonstrated robust TrkB phosphorylation in primary neurons (Fig 1A). Specifically, analog **5**, HIFN, a fluoropyridine derivative of HIOC (Fig 1D), exhibited heightened TrkB activity in NIH-3T3-TrkB cells in comparison to the parent molecule HIOC at a concentration of 10 nM (Fig 1B and 1C).

### HIFN *vs* HIOC: enhanced protection in ocular trauma

In our earlier study of HIOC, we demonstrated the therapeutic potential of TrkB modulators in mitigating ocular trauma-induced visual dysfunction [1]. Prompted by our cell-based screening, we investigated the protective effects of analogs **5** and **6** against trauma injury in a mouse model of blast overpressure injury [17]. Surprisingly, analog **6**, irrespective of *in-vitro* TrkB activation, showed no statistically significant protection against vision loss in blast-exposed mice (S7 Fig in S1 File). In contrast, analog **5** (HIFN) effectively mitigated damage caused by overpressure injury as described below.

**Fig 2:**
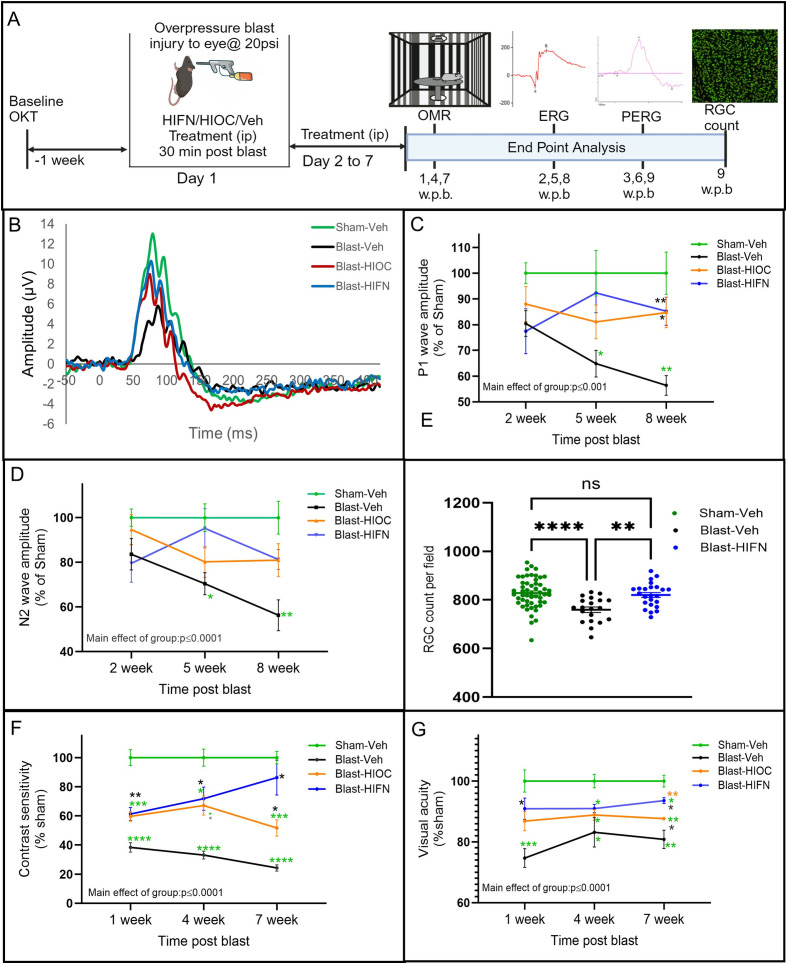
HIFN reduces retinal and visual function deficits following ocular trauma. (A) Schematic of experimental timeline. Baseline OMR was recorded in animals one week before trauma induction. The animals were randomly divided into various groups. On day 1, overpressure injury was induced by a 20 psi air blast; 30 minutes after injury, animals were administered the first dose of vehicle, HIFN (40 mg/kg ip), or HIOC (40 mg/kg ip). The animals were treated for another 6 days with daily ip injections. OMR, scotopic ERG and PERG were recorded at different timepoints as indicated in the figure; week post blast (w.p.b). (B) Representative PERG waveform from each group. (C and D) Pattern ERG was recorded for RGC function assessment at various times post-blast. P1 (C) and N2 (D) wave amplitudes begin to decline progressively over the 8 weeks. HIFN treatment prevented the decline in RGC cell function and preserved the P1 and N2 amplitude at 8 w.p.b. E) Retinal wholemounts were immunostained for the RGC -specific marker RBPMS. Cells in ROI were automatically counted using ImageJ, Simple RGC plugin. RGC cell count decreases after blast in vehicle-treated mice while, HIFN treatment prevented the RGC cell loss. Contrast sensitivity (F) and visual acuity (G) at 1,4, and 7weeks post blast. Data is presented as % of Sham-Veh ± SEM. HIFN rescued visual acuity and contrast sensitivity deficit at all the time points. At 7 weeks, visual function is better in HIFN-treated animals compared to HIOC. (C) and (D) * p ≤ 0.05 vs Sham-Veh, ** p ≤ 0.01 vs Sham-Veh; * p ≤ 0.05 vs Blast-Veh; ** p ≤ 0.01 vs Blast Veh; n = 5–7per group. Data are expressed as %Sham-Veh ± SEM; REML, Holm Sidak’s adjusted p-values used for post-hoc comparisons. (E) Data are expressed as total RBPMS-positive cells/ROI (mean ± SEM) ** p = 0.002; **** p ≤ 0.0001; n = 20–56. (F) and (G) * p ≤ 0.05 vs Sham-Veh, ** p ≤ 0.01 vs Sham-Veh, ***p ≤ 0.001 vs Sham-Veh, ****p ≤ 0.0001 vs Sham-Veh; * p ≤ 0.05 vs Blast-Veh, ** p ≤ 0.01 vs Blast-Veh; ** p ≤ 0.01vs Blast-HIOC; n = 6–7per group; REML, Holm Sidak’s adjusted p-values used for post-hoc comparisons.

For *in-vivo* studies, animals were randomly assigned to Sham-Vehicle (Veh), Blast-Veh, Blast-HIOC, and Blast-HIFN groups. Contrast sensitivity and visual acuity recordings, a week before blast, revealed no significant intergroup baseline differences. Animals were administered a dose of 40 mg/kg intraperitoneally (ip) of HIFN or HIOC, unless specified otherwise. Treatment was initiated on the day of blast exposure and continued daily for one week (Fig 2A).

To understand which retinal cell types are affected by blast injury, we recorded electroretinograms (ERG) to map the functional activity of retinal cells. Full-field scotopic ERG recordings (–3.0 to 1 log cd^.^s/m^2^) at 3, 6, and 9 weeks after blast showed no observable difference for any ERG component across time (S2 Table in S1 File). No significant differences were observed in photoreceptor and bipolar cell function (S8A, S8B Fig in S1 File) as analyzed by ‘a-wave’ (main group effect, F (3, 110) = 1.1.3, p = 0.340) and ‘b-wave’ amplitudes (main group effect, F (3,110) = 0.848, p = 0.470). We further isolated oscillatory potentials (OPs) and ‘c-wave’ amplitudes from ERGs at 9 weeks. OPs arise from amacrine cell responses on the ascending limb of the ‘b-wave’ while the ‘c-wave’ indicates retinal pigment epithelial cell function. We observed no alterations in ERG ‘c-wave’ amplitudes in any treatment group (p = 0.244; S8C Fig in S1 File), suggesting normal functioning of retinal pigment epithelial cells. Similarly, the amplitudes of OPs were unaltered after the blast exposure (p = 0.117; S8D Fig in S1 File). These findings indicated that ocular trauma under the conditions used in our study did not significantly impair photoreceptor, bipolar cell, or amacrine cell light responses.

Traumatic injuries often affect retinal ganglion cells, given the vulnerability of their axons as they extend long distances to brain centers [20]. Since the function of outer or inner retinal neurons appeared intact, we next examined the functionality of RGCs using the PERG. PERG revealed significant changes in both P1 and N2 wave amplitudes [2-way, mixed model ANOVA, {P1 amplitude main group effect F (3,21)=11.21, p ≤ 0.0001}; {N2 amplitude, main group effect F (3,81) =11.80, p = 0.0001}]. In blast-exposed animals treated with vehicle, both P1 and N2 amplitudes progressively declined over 8 weeks (Figs 2B-2D). Initial assessment 2 weeks after blast did not show any significant differences between blast-exposed and sham animals. However, 5 weeks after blast, both P1 (p = 0.03, vs Sham-Veh) and N2 wave amplitudes (p = 0.017, vs Sham-Veh) decreased in vehicle-treated animals. RGC function appeared better in animals with HIFN treatment as reflected by P1 amplitude, but the effect was not statistically significant (p = 0.07 vs Blast-Veh). A further assessment of RGC function at 8 weeks post-blast confirmed a continued decline in the vehicle treatment group (p = 0.007 for P1 amplitude; p = 0.005 for N2 amplitude vs Sham-Veh), suggesting a slow but progressive decline in RGC cell function after blast. At this time point, both HIFN and HIOC rescued the deficit in RGC cell function as evidenced by significantly higher P1 wave amplitudes in HIOC (p = 0.01 vs Blast-Veh) and HIFN (p = 0.007 vs Blast-Veh) treated mice. Moreover, N2 wave amplitude modestly improved in drug-treated animals (p = 0.051, HIFN vs Veh).

To correlate the RGC function with the cell number, we prepared retinal whole-mounts from animals sacrificed after PERG recordings and stained them for cell-specific markers of RGCs. Four regions of interest (ROI) were selected from each whole-mounted retina (400 μm from the optic disc) for quantification of RGC number using two markers, RBPMS and Brn3a. There were significantly fewer RBPMS-positive cells in the Blast-Veh group compared to Sham animals (p ≤ 0.001; Fig 2E); HIFN treatment prevented this decline in RGC after blast (Fig 2E). Blast-HIFN animals had a significantly (p = 0.002) higher number of RGCs than vehicle-treated animals. Quantification of Brn3a+ cells also revealed a significant loss of RGCs in the retina of Blast-Veh mice compared to Sham (p = 0.019; data not shown); Blast-HIFN mice showed no significant decline in RGCs compared to Sham and a trend for an increased number of RGCs compared to Blast-Veh mice (p = 0.09). Such differences in Brn3a and RBPMS labelling have been previously reported [21], probably related to Brn3a not labeling all RGC subtypes.

Blast exposure significantly impacted visual function outcomes, assessed by the optomotor response (OMR), with a decline in contrast sensitivity evident as early as 1 week after blast exposure, when it was reduced to 38.4 ± 3.2% (p ≤ 0.0001) of sham control animals (Fig 2F). Contrast sensitivity continued to decline (24.3 ± 2.1%, p ≤ 0.0001) with time until 7 weeks post-blast (the longest time point recorded). Preservation of contrast sensitivity with either HIOC (59.6 ± 3.2%, p = 0.002 vs Veh) or HIFN (61.3 ± 4.5%, p = 0.007 vs Veh) was observed at 1-week post-blast. By 4 weeks, contrast sensitivity declined by 67% in the vehicle group, while a decline in animals treated with HIOC (33%, p ≤ 0.01 vs Veh) and HIFN (39%, p ≤ 0.01 vs Veh) was significantly less. Notably, at 7 weeks after blast, contrast sensitivity of HIFN-treated mice was 3-fold higher than that of vehicle-treated mice and was not significantly different than that of sham controls (p = 0.32) (Fig 2F). Moreover, HIFN-treated animals demonstrated a 22.3% higher (p = 0.06 vs HIOC) contrast sensitivity than the HIOC group at 7 weeks after blast.

Visual acuity deficits, as assessed by spatial frequency threshold in OMR, began at 1 week post-blast (Fig 2G) in parallel to lower sensitivity to contrast, although compared to contrast sensitivity, visual acuity showed less sensitivity to blast. Visual acuity showed a 25.2% decline one-week post-blast (p ≤ 0.001 vs sham). HIOC and HIFN treatment resulted in better visual acuity preservation compared to the vehicle group. 7 weeks after blast, the visual acuity was 8.3% higher with HIOC (p = 0.06) and 16% with HIFN (p ≤ 0.01) compared to vehicle-treated animals. Furthermore, HIFN was significantly better than HIOC in reducing the visual acuity deficit (p = 0.002). Together, these results indicate that BDNF mimetics can prevent the functional decline in retinal function and provide better vision outcomes after ocular injury. Moreover, HIFN exhibited long-lasting protective effects, superior to the parent molecule HIOC in preventing vision loss due to traumatic injury.

The comprehensive study to evaluate the protective effects and efficacy of HIFN was initially performed on male C57BL6/J mice. To determine if these effects were sex-specific, trauma was induced in female C57BL6/J mice as well (S9 Fig in S1 File). Visual acuity deficits were recorded in both HIFN-treated and non-treated males and females 8 days after blast. The treatment with HIFN reduced the visual acuity decline in both sexes to a comparable degree (S9 Fig in S1 File).

### Neuroprotection by HIFN is TrkB-dependent

**Fig 3:**
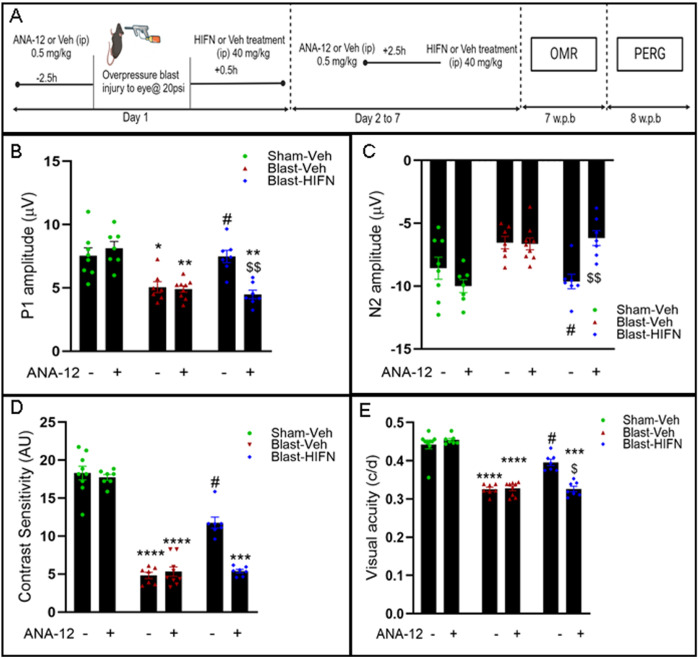
HIFN protection of vision loss is Trk-B dependent: (A) Schematic representation of the experimental protocol of ANA-12 and HIFN treatment. ANA-12, a specific TrkB receptor inhibitor, was used to block TrkB activity. On day 1, animals received 0.5 mg/kg of either ANA-12 or vehicle 2.5 h prior to blast injury. Animals were administered HIFN or vehicle 30 min after blast exposure. ANA-12 pretreatment followed by vehicle/HIFN was continued for a week. (B) and (C) PERG was recorded at 8 w.p.b.; preservation of P1 and N2 wave amplitude by HIFN was not seen in animals that were pretreated with ANA-12. (D) and (E) Contrast sensitivity and visual acuity were recorded at 7 w.p.b.; Contrast sensitivity and visual acuity decline in HIFN-treated animals were seen when animals were pretreated with ANA-12. *p≤0.05, **p≤0.01, ***p≤0.001, ****p≤0.0001 vs Veh/Sham-Veh; #p≤0.05 vs Veh/Blast-Veh; $p≤0.01vs Veh/Blast-HIFN, $$p≤0.01vs Veh/Blast-HIFN; n=7-9/group.

HIFN potently activated the TrkB receptor (Fig 1), suggesting that the preservation of RGC and visual function is conferred via TrkB-mediated signaling. To test this hypothesis, on day 1, 2.5 h before the blast, animals received a systemic injection of ANA-12 (0.5 mg/kg) or its vehicle. ANA-12 is a non-competitive inhibitor of TrkB that selectively blocks its activation [22]. Subsequently, 30 minutes after the blast, animals were treated with HIFN or vehicle. Additionally, from day 2 to 7, ANA-12/vehicle was injected 2.5h before HIFN/vehicle treatment to interfere with the TrkB activation (Fig 3A). The study aimed to assess the impact of ANA-12 pretreatment on the neuroprotective effects exerted by HIFN.

As discussed above, HIFN reduced the loss of RGC function induced by traumatic injury (Figs 2B-2D). Pretreatment with ANA-12 eliminated these protective effects of HIFN on RGC function (Figs 3B and3C). ANA-12 alone had no significant effect on the P1 amplitude of the PERG in the Sham mice (p = 0.99) or on the reduction in amplitude following blast (p = 0.99) (Fig 3B). However, ANA-12 completely blocked the protective effect of HIFN administration; the P1 amplitude in the ANA-12 pretreatment group (ANA-12/Blast-HIFN) was significantly lower (p = 0.003) compared to the group not treated with ANA-12 prior to HIFN (Veh/Blast-HIFN), and not significantly different from the ANA-12-treated group not administered HIFN. Likewise, N2 amplitude showed a similar pattern (Fig 3C) where ANA-12 had no effect alone (Sham, p = 0.65; Blast, p = 0.97), but blocked the increased amplitude caused by HIFN (p = 0.008).

Additionally, ANA-12 blocked visual function improvements with HIFN (Figs 3D and 3E) in mice exposed to blast injury. Similar to RGC function, ANA-12 alone had no effect on contrast sensitivity or visual acuity in Sham or blast-exposed mice. HIFN reduced the contrast sensitivity deficit in mice not pretreated with ANA-12 (Blast-Veh vs Blast-HIFN, p = 0.02), but not in those with ANA-12 pre-treatment. Contrast sensitivity in mice treated with ANA-12 and HIFN was comparable to that in mice treated with ANA-12 alone (p = 0.61). Visual acuity threshold revealed the same pattern as contrast sensitivity. Animals treated with HIFN following blast exposure have significantly (p = 0.017) better visual acuity threshold than vehicle-treated mice. The improvement in visual acuity with HIFN treatment was not seen in ANA-12/Blast-HIFN animals. The mice treated with ANA-12 and HIFN had visual acuity similar to the mice treated with ANA-12 alone and significantly lower (p = 0.026) than the HIFN alone group. Taken together, the data showed that pharmacological inhibition of TrkB activation blocked the protection exerted by HIFN, supporting our hypothesis that HIFN acts via TrkB receptor-mediated signaling.

### Neuroprotective effects of HIFN are dose-dependent

**Fig 4:**
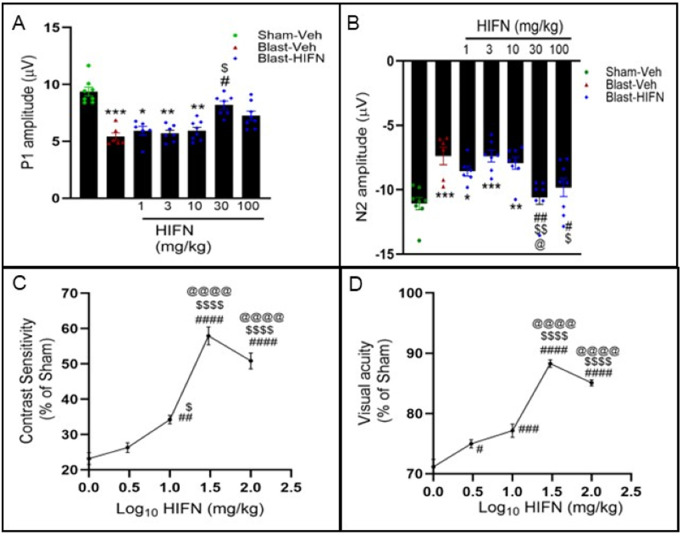
Dose-dependent neuroprotection by HIFN. Various doses of HIFN were tested to find the effective dose. (A) PERG P1 wave and (B) PERG N1 wave recorded at 8 weeks post-blast. (C) Contrast sensitivity and (D)Visual acuity recorded at 7 weeks pots-blast. The best responses were observed with 30 mg/kg for reducing RGC and visual function dysfunctions. *p≤0.05, **p≤0.01, ***p≤0.001, ****p≤0.0001 vs Sham-Veh; #p≤0.05, ##p≤0.01, ####p≤0.0001 vs Blast-Veh, $p≤0.05, $$p≤0.01, $$$$p≤0.0001 vs 3mg/kg group; @p≤0.05, @@@@p≤0.0001 vs 10mg/kg group; n= 6-8/group.

All our experiments were conducted with 40 mg/kg of HIFN, which was the optimal dose found for HIOC in our previous study [1]. Aiming to identify the optimal dosage for achieving neuroprotection with HIFN, we next conducted a dose-response study. HIFN was injected into animals at doses ranging from 1 to 100 mg/kg. A 30 mg/kg dose significantly reduced the decline in P1 (vs Blast-Veh p = 0.013) and N2 (Blast-Veh, p = 0.004) wave amplitudes 8 weeks after blast exposure (Figs 4A and 4B). Animals treated with 1 mg/kg, 3 mg/kg, and 10 mg/kg HIFN following blast-exposure showed diminished RGC function similar to vehicle-treated animals, indicating that these lower doses were ineffective in rescuing RGC damage. The highest dose tested (100 mg/kg) had significantly improved N2 amplitude (vs Blast-Veh, p = 0.04), but improvement in P1 amplitude, though better than vehicle treatment, did not reach statistical significance (p = 0.32).

We recorded dose-dependent preservation of visual function 7 weeks after blast exposure, with an apparent peak dose of 30 mg/kg (Figs 4C and 4D). We saw significant improvement in contrast sensitivity (Fig 4C) starting from a dose of 10 mg/kg (vs Blast-Veh, p = 0.008) and continuing to improve with higher doses of 30 mg/kg and 100 mg/kg (vs Blast-10 mg/kg, p ≤ 0.0001). Likewise, visual acuity increased significantly from 71.2 ± 1.2% of sham control to 85.1 ± 0.5% (p = 0.0001) with HIFN dose increasing from 1 mg/kg to 100 mg/kg HIFN (Fig 4D). An HIFN dose as low as 3 mg/kg was effective in visual acuity preservation compared to vehicle (p = 0.019) (Fig 4D). Maximum visual acuity preservation (88.3 ± 0.64% of sham control) was observed with the 30 mg/kg dose (vs Blast-Veh, p ≤ 0.0001). Overall, these results show a dose-dependent neuroprotection by HIFN. Acuity and contrast preservation were achieved starting with a dose of 10 mg/kg, but RGC function improvement required a higher dose. In summary, in our experiments, animals treated with 30 mg/kg had the best visual outcomes.

### Therapeutic time window for HIFN-induced neuroprotection is limited to 3 h after injury

**Fig 5:**
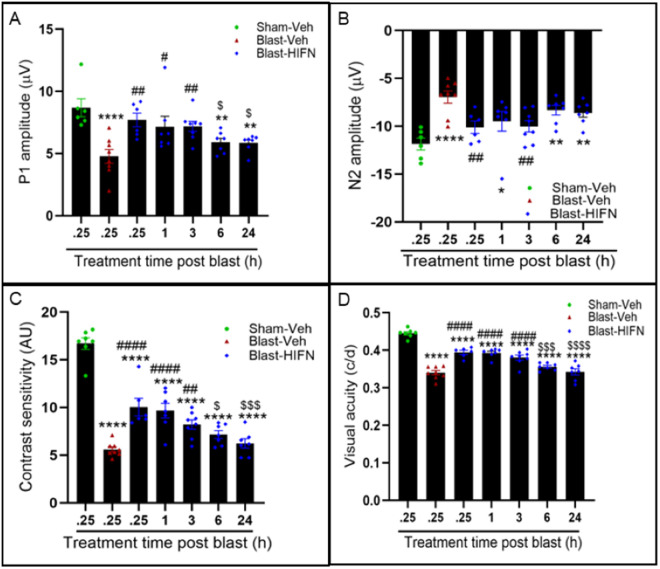
Critical time window for HIFN intervention: Mice received their initial treatment with HIFN (40 mg/kg) at various times relative to blast exposure (treatment time post blast) and were treated daily for the next 6 days. Initial treatment was provided as early as 15 minutes (0.25 h) after the blast and delayed up to 24 h. PERG P1-wave amplitude (A) and N2-wave amplitude (B), contrast sensitivity (C), and visual acuity (D) were recorded. HIFN reduced vision loss with initial treatments up to 3 h, but was less effective at 6 h or 24 h.*p ≤ 0.05, ^**^p ≤ 0.01, ^****^p ≤ 0.0001 vs Sham-Veh; ^**#**^p ≤ 0.05, ^**##**^p ≤ 0.01, ^**####**^p ≤ 0.0001 vs Blast-Veh; ^**$**^p ≤ 0.05, ^**$$$**^p ≤ 0.001, ^**$$$$**^p ≤ 0.0001vs 0.25h; n = 6-8/group.

To determine the therapeutic treatment window for HIFN, we injected the initial dose of HIFN at different time intervals after blast. Mice received 40 mg/kg of HIFN starting at 0.25, 1, 3, 6 or 24 h after blast exposure; mice then received daily injections at the same time of day for an additional 6 days. We recorded changes in visual function and RGC function at 7 weeks and 8 weeks post blast, respectively. HIFN treatment mitigated vision loss if provided within 3 h of injury (Fig 5). Beyond the 3 h time window, HIFN loses its efficacy in rescuing the vision decline.

There was improved function of RGC cells as recorded by PERG in animals treated with HIFN within a time window of 3 h after injury. P1 amplitude (p ≤ 0.001, p = 0.02, p = 0.002 for 0.25 h, 1 h, and 3 h, respectively, vs Blast-Veh) and N2 amplitude (p = 0.004, p = 0.06, p = 0.002 for 0.25 h, 1 h, and 3 h, respectively, vs Blast-Veh; Figs 5A and 5B) were significantly higher in animals treated with HIFN. RGC function was not rescued if the initial HIFN treatment was delayed for 6 hours or more. Visual function data supported PERG results. HIFN treatment preserved contrast sensitivity even if the treatment was delayed up to 3 h (vs Blast-Veh, p = 0.014). However, providing treatment within 1 h gave the best response. Contrast sensitivity was 60.7 ± 5.5% (p ≤ 0.0001) for 0.25 h and 57.9 ± 4.6%, (p ≤ 0.0001) for the 1 h group compared to 33.6 ± 1.57% for vehicle-treated animals (Figs 5C and 5D). A treatment window not exceeding 3 h reduced visual acuity deficits. Visual acuity was significantly better than the Blast-Veh group in animals treated at 0.25 h (p ≤ .0001), 1 h (p ≤ 0.0001), and 3 h (p ≤ 0.0001) after blast. If the first dose of HIFN was administered 6 or 24 h after blast exposure, the visual acuity deficit was not significantly reduced.

### Promising safety profile of HIFN in toxicity testing

The safety profile of HIFN was assessed using acute and chronic toxicity studies in both male and female animals. For acute toxicity studies, animals were subjected to a dose of 300 mg/kg and monitored for toxicity, including changes in fur color, lethargy, red secretions around the eye, hunch back, tremors, as well as mortality for a 7-day period. Remarkably, at this dose, no mortality or observable signs of toxicity were noted. Subsequently, a higher dose of 600 mg/kg was administered to another cohort of animals. Even at this elevated level, 20 times the maximal effective dose for visual function protection, all animals remained active, displaying no visible signs of toxicity.

**Fig 6:**
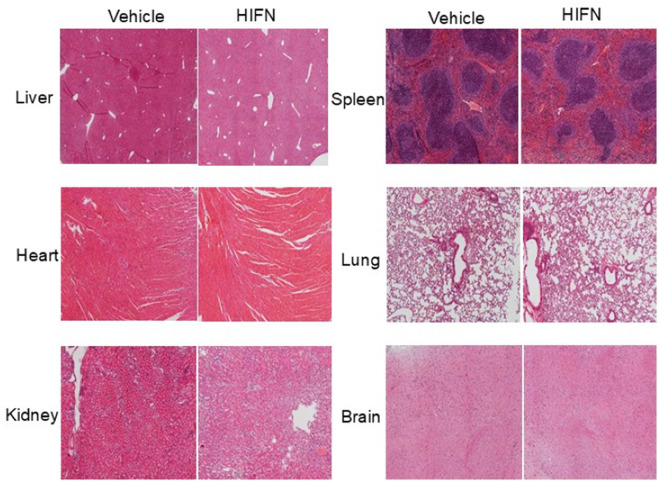
No signs of toxicity of HIFN treatment were reported in the histopathological analysis. Representative images of hematoxylin/eosin-stained tissue sections from animals treated with HIFN or vehicle for 40 days. The sections were analyzed by a board-certified pathologist (HEG). No abnormal changes due to HIFN were observed. n = 3/group.

For the evaluation of chronic toxicity, male and female mice received a daily dose of 40 mg/kg HIFN for a duration of 40 days. Body weights of vehicle and HIFN-treated mice were not significantly different at baseline or after treatment for 40 days (baseline males: Veh 30.0 ± 0.6 g, HIFN 29.8 ± 0.6 g; baseline females: Veh 18.8 ± 0.3 g, HIFN 18.8 ± 0.6 g; post-treatment males: Veh 29.5 ± 0.5 g, HIFN 29.7 ± 0.3 g; post-treatment females: Veh 19.2 ± 0.3 g, HIFN 18.6 ± 0.4 g). Blood and serum samples were collected for complete blood count and serum biochemistry analyses. Brain, liver, kidney, spleen, and heart were harvested for histopathological examination of sections. In male mice, we observed no significant differences in blood parameters or liver and kidney function tests between the control and HIFN-treated groups (Table 1 and Table 2). HIFN-treated females exhibited an increase in the number of white blood cells and lymphocytes (Table 1); however, aside from these changes, all other parameters remained comparable to the control group (Tables 1 and 2). Importantly, there were no evident signs of histopathological toxicity upon analysis of stained tissue sections (Fig 6). In conclusion, the results from both chronic and acute toxicity studies indicate that HIFN is relatively non-toxic. The absence of mortality or observable signs of toxicity at high doses in acute toxicity assessments, coupled with the lack of significant adverse effects on various physiological parameters and organ histopathology in chronic toxicity studies, supports the safety profile of HIFN. These findings collectively suggest that HIFN holds promise as a safe therapeutic agent.

**Table1:** CBC (complete blood count) profile of male and female animals treated chronically with HIFN or vehicle.

Blood Test	Male			Female		
	Vehicle	HIFN	p-value	Vehicle	HIFN	p-value
White blood cells (per liter)	1.52*10^9^	2.46*10^9^	0.081	1.11*10^9^	2.03*10^9^	0.004
Lymphocytes (per liter)	1.36*10^9^	2.25*10^9^	0.082	1.03*10^9^	1.92*10^9^	0.003
Monocytes (per liter)	0.053*10^9^	0.056*10^9^	0.90	0.03*10^9^	0.04*10^9^	0.61
Neutrophils (per liter)	0.103*10^9^	0.151*10^9^	0.53	0.05*10^9^	0.07*10^9^	0.56
Lymphocyte (%)	89.72	91.85	0.48	91.87	94.85	0.10
Monocyte (%)	3.52	2.47	0.57	2.88	1.93	0.25
Neutrophils (%)	6.78	5.68	0.73	5.25	3.20	0.24
Red blood cells (per liter)	8.28*10^12^	8.25*10^12^	0.99	8.88*10^12^	8.05*10^12^	0.14
Hemoglobin (g/liter)	10.73	10.68	0.90	11.95	10.55	0.09
Hematocrit (%)	34.87	35.31	0.72	37.56	34.12	0.17
Mean corpuscular volume (fL)	42.17	42.83	0.31	42.00	42.33	0.87
Mean corpuscular Hb (pg)	12.93	12.93	0.99	13.45	13.12	0.21
Mean corpuscular Hb conc (g/liter)	30.75	30.20	0.053	32.00	30.92	0.06
RDWc (%)	19.55	19.98	0.28	20.10	20.45	0.26
RDWs (fL)	30.72	32.17	0.09	31.92	32.55	0.47
Platelet (per liter)	170.33*10^9^	276.5*10^9^	0.30	340.00*10^9^	298.33*10^9^	0.69
Mean platelet volume (fL)	8.08	6.70	0.73	6.30	7.52	0.67
Plateletcrit (%)	0.12	0.18	0.28	0.22	0.19	0.32
PDWc (%)	25.80	27.33	0.96	28.22	25.10	0.20
PDWs (fL)	8.83	8.17	0.62	8.28	8.07	0.48

n=6 animals/group.

Abbreviations: RDWc: RBC distribution width, coefficient of variation; RDWs: RBC distribution width, standard deviation; PDWc: Platelet distribution width, coefficient of variation; PDWs: Platelet distribution width, standard deviation; fL: femptoliter.

**Table 2:** Serum chemistry in the animals after chronic treatment with HIFN or vehicle.

Test	Male			Female		
	Vehicle	HIFN	p-value	Vehicle	HIFN	p-value
ALB (g/dL)	2.44 ± 0.24	2.56 ± 0.07	0.80	2.7 ± 0.1	2.55 ± 0.1	0.33
ALP (U/L)	37.6 ± 6.4	39.6 ± 1.3	0.297	70.3 ± 5.5	64.2 ± 2.53	0.332
ALT (U/L)	47.6 ± 8.8	58.8 ± 9.5	0.412	64.3 ± 18.5	61.7 ± 4.9	0.89
AMYL (U/L)	837 ± 224	669 ± 25	0.478	624 ± 28	553 ± 43	0.08
AST (U/L)	533 ± 126	473 ± 81	0.690	467 ± 131	510 ± 90	0.79
BUN (mg/dl)	29.6 ± 4.0	26 ± 3.6	0.53	22.3 ± 1.5	19.5 ± 1.1	0.339
CREAT (mg/dl)	0.272 ± 0.05	0.424 ± 0.03	0.051	0.35 ± 0.04	0.43 ± 0.04	0.206
DBILI (mg/dl)	0.44 ± 0.16	0.44 ± 0.07	0.99	0.36 ± 0.08	0.35 ± 0.06	0.99
LDH (U/L)	1414 ± 317	1259 ± 198	0.68	1037 ± 294	1216 ± 1943	0.62
TBILI (mg/dl)	0.556 ± 0.12	0.476 ± 0.05	0.54	0.413 ± 0.06	0.49 ± 0.07	0.461

n= 6 animals/group.

Abbreviations: ALB: albumin; ALP: Alkaline phosphatase, ALT: alanine transaminase; AMYL: Amylase; AST: aspartate transaminase; BUN: blood urea nitrogen; CREAT: creatinine; DBILI: direct bilirubin, LDH: lactate dehydrogenase; TBILI: total bilirubin.

## Discussion

Lack of effective treatment for traumatic eye injuries has encouraged extensive research into the mechanisms underlying progressive damage resulting from these injuries. Numerous studies provide evidence supporting the involvement of RGC death [20] and TrkB signaling in blast-related injuries [23–25]. A positive correlation is established between BDNF/TrkB levels and functional outcomes post-injury. However, the pharmacokinetic constraints of BDNF limit its therapeutic use. Recently, nanoparticle-bound BDNF, which readily cross the blood-brain barrier, demonstrated effectiveness in treating traumatic blast injury [26], highlighting the importance of TrkB signaling in such injuries. Recognizing the potential of TrkB agonists, synthetic small molecules such as 7,8-dihydroxyflavone (DHF) and LM22A4 have shown promise in attenuating injury-related pathology [14]. Our previous study showcased the efficacy of a small molecule, HIOC (**1**), a synthetic derivative of N-acetyl serotonin, in preventing blast-induced vision loss in mice [1]. Here, we introduce HIFN (**5**), demonstrating improved efficacy over HIOC in diminishing progressive vision loss following ocular trauma. HIFN is an achiral analog of chiral HIOC, having a fluoropyridine group in place of the six-membered lactam ring of HIOC. Individual enantiomers of HIOC are not configurationally stable, due to the position of the chiral center between two carbonyl groups, which promotes facile enolization (S1 and S2 Figs in S1 File). Although each enantiomer of HIOC may exhibit distinct levels or types of bioactivities, the achiral nature of HIFN provides another advantage for HIFN over HIOC as a therapeutic agent.

A decline in contrast sensitivity and visual acuity is an established marker of traumatic eye injury [17,27]. Our study, utilizing the same overpressure blast model, corroborated these findings by demonstrating a continuous decline in spatial frequency thresholds and contrast threshold in blast-exposed animals (Fig 2). Both HIOC and HIFN preserved the contrast sensitivity and visual acuity in animals for at least two months after the blast. Notably, the protection afforded by HIFN surpassed that of HIOC, making it a promising candidate for further investigations.

PERG, a functional readout of retinal ganglion cells, revealed that blast injury primarily affected RGCs (Figs 2 and 3). Our results align with other studies reporting alterations in RGC function following injury [28]. The retina is a complex structure wherein intermediary bipolar cells pass on the signal generated in photoreceptors in the outer retinal layers to the retinal ganglion cells. Any damage to outer or inner neurons also disrupts signaling to RGCs and affects their responses. Unlike some studies reporting subnormal [29] and supernormal [27] ERG responses post-injury, our scotopic ERG data did not reveal involvement of outer or inner nuclear layer neurons. These results were consistent with our previous study, where amplitudes of ERG a- and b-waves were unaltered after the blast injury [1]. This discrepancy could be attributed to several procedural factors including distance of the eye from the blast source, pressure of blast wave, the type of driver gas used (helium, nitrogen, etc.), and animal species/strain [30]. In our model, blast overpressure injury significantly decreased the RGC function without affecting ERG a- or b-waves. The preservation of PERG function with HIFN suggests a protective effect on RGCs. The decrease in RGC function following blast is also consistent with the blast-induced decrease in contrast sensitivity, which was reversed by HIFN. Blast caused a small but significant decrease of RGC number, which was also mitigated by HIFN. Considering the large decrements of contrast sensitivity and reversal by HIFN, the changes in RGC cell number were remarkably small. The reasons for this apparent discrepancy are unclear, but may involve changes in the optic nerve, such as disruptions of myelination or rewiring in synaptic terminal regions, that did not cause RGC cell death. Further studies involving optic nerve recording or analyzing synaptic markers and targets may provide valuable insight into the mechanisms of vision preservation by HIFN.

Our investigation into the underlying neuroprotective mechanism of HIFN revealed its dependence on TrkB receptor activation. The administration of a specific TrkB receptor blocker, ANA-12 [22], negated the protective effect of HIFN on vision loss. This was not surprising, as HIOC, the parent analog of HIFN, also exerts its protection through TrkB signaling [1,31]. Although HIFN was given for only the first week after blast exposure, *the protection was sustained or improved over the subsequent two months*. TrkB/BDNF signaling is involved in neuronal survival and plasticity. We propose that HIFN has promoted neuronal survival and aided surviving neurons in making new connections, by activating TrkB.

The preservation of visual function was dose-dependent, with an apparent maximal response at 30 mg/kg. Interestingly, the highest tested dose (100 mg/kg) showed a marginal decline in protective effects, potentially hinting at off-target binding issues at higher concentrations. Off-target binding may stimulate other unknown pathways. The critical therapeutic window in response to traumatic blast injury for HIFN was identified as 3 hours, beyond which the neuroprotective effects were not realized. A plausible reason is that irreversible damage may have occurred at latencies beyond this time.

The present study not only confirms that HIFN modulates TrkB receptor activation but also provides evidence of its efficacy in preserving vision decline resulting from traumatic injuries. A limitation of our study is that the exact mechanism by which a small molecule, particularly one with a molecular weight of 300 Daltons, fits into the active site of the TrkB dimer and triggers its activation remains unknown. Current hypotheses suggest that small molecules like HIFN may interact with a TrkB monomer, inducing structural changes in the receptor that facilitate dimer formation [2]. Alternatively, HIFN may act on preformed TrkB dimers [32]. In this scenario, small molecules could bind to these preexisting dimers, altering their conformation and promoting phosphorylation, which is a key step in receptor activation.

BDNF and serotonin pathways often function in a cooperative manner to regulate neuronal plasticity and survival. Serotonin receptor activation by antidepressants and psychedelics indirectly increases BDNF. Some of the psychedelics bind to TrkB, inducing allosteric modulation [33]. Another possibility is that HIFN binds to serotonin receptor, causing indirect activation of TrkB by elevating BDNF. However, it was previously shown that NAS activates TrkB in a BDNF-independent manner and that neither serotonin nor its metabolite, 5-hydroxyindole acetic acid, directly stimulates TrkB phosphorylation in vitro [34]. HIFN, being derived from NAS, is therefore likely to exhibit these properties; however, activation of serotonin receptor or other receptors indirectly affecting TrkB cannot be excluded without future studies. The intricacies of how these small synthetic molecules precisely bind to and activate the TrkB receptor are areas of ongoing research. Future studies will likely delve into the details of the molecular interactions, the specific binding sites involved, and the conformational changes induced by these small molecules in the context of TrkB receptor modulation.

Our ERG data indicate that outer retinal neurons were not affected; however, this assessment does not account for glial cells within the retina. Glial cells are key responders to retinal injury and can play either protective or detrimental roles. Specifically, microglia can adopt a pro-inflammatory phenotype that drives secondary injury, or alternatively, an anti-inflammatory state that promotes debris clearance and tissue repair [35]. Notably, BDNF has been shown to suppress pathogenic microglial activation [36]. How these glial responses are regulated in the current context, and whether HIFN treatment influences their behavior, remains an important question for future investigation. Understanding these mechanisms will not only contribute to our knowledge of neuroprotection but may also pave the way for the development of targeted therapies for neurodegenerative disorders.

In summary, HIFN is a promising TrkB receptor activator, as a potential alternative to BDNF therapy in the realm of treating neurodegenerative diseases. The demonstrated efficacy of HIFN in preserving vision and preventing progressive damage following traumatic injury suggests its potential utility in addressing a wide range of neurological and psychiatric disorders characterized by compromised BDNF/TrkB signaling.

## Supporting information

S1 File Supplementary figures and tables. (PDF)
