# Supplementary material for: Long-term functional rescue of trauma-induced vision loss by a novel, small molecule TrkB modulator
Source: PLoS One. 2025 Sep 29;20(9):e0320231. doi: 10.1371/journal.pone.0320231 (PMC12478906; doi:10.1371/journal.pone.0320231)
Supplement: S1 File — (PDF) [file pone.0320231.s001.pdf]

Supporting Information for:
Long-Term Functional Rescue of Trauma-Induced Vision Loss by a Novel, Small Molecule TrkB Activator

Shweta Modgil, Christopher L. Walker, Micah A. Chrenek, Hans E. Grossniklaus, Frank E. McDonald, P. Michael Iuvone

P. Michael Iuvone
Email: miuvone@emory.edu; frank.mcdonald@emory.edu

This PDF file includes:

Supporting text
Figures S1 to S9
Table S1 and S2
SI References

Chemical Synthesis of Analogs

General Experimental

^{1}H and ^{13}C nuclear magnetic resonance (NMR) spectra were recorded with a Varian AVIII 400 spectrometer and an AVANCE 600 spectrometer equipped with a cryogen probe. NMR spectra were measured from solutions in deuterated dimethylsulfoxide ($(CD_3)_2SO$, using the residual solvent resonance as internal standard, $(CD_3)_2SO$: 2.50 ppm for ^{1}H NMR and 39.5 ppm for ^{13}C NMR, and were reported in parts per million (ppm). Abbreviations for NMR signal coupling are as follows: s, singlet; d, doublet; t, triplet; q, quartet; dd, doublet of doublet; ddd, doublet of doublet of doublet; m, multiplet; br, broad.

Mass spectra (high resolution ESI and APCI) were recorded on a Scientific Exactive Plus Mass spectrometer, using the orbitrap mass analyzer, which is an electrostatic ion trap. Crystal structures were obtained with an XtaLAB Synergy-S diffractometer equipped with an Oxford Cryosystems low-temperature device.

Thin layer chromatography (TLC) was performed on a precoated glass backed plates purchased from Silicycle (silica gel 60F254; 0.25 mm thickness), or on precoated aluminum-backed plates purchased from Whatman (silica gel 60F254). Flash column chromatography was carried out with silica gel 60 (230-400 mesh ASTM) from Silicycle.

All reactions were conducted with anhydrous solvents in oven-dried glass vials or flasks, under argon atmosphere. Reactants were used as received from commercial suppliers without prior purification, as were solvents used for extractions and chromatographic separations. All chemicals were purchased from Sigma Aldrich, Oakwood Chemical, or TCI Chemicals. Only carbonyldiimidazole (CDI) purchased from Sigma Aldrich gave reliable results in the *N*-acylation synthetic reactions. Anhydrous CH_2Cl_2 was obtained from a Pure Process Purification solvent system.

General synthetic method

The synthesis of HIOC analogs generally followed the *N*-acylation protocol developed for the synthesis of HIOC (**1**) from serotonin hydrochloride (**7**) and the *delta*-valerolactam carboxylic acid (**8**) (**S1 Fig**).[1] These optimized conditions favored selective acylation of the primary amine of serotonin hydrochloride via *N*-acylimidazole intermediates. Analog **2**, with a methyl substituent between the carbonyl groups, cannot undergo configurational interconversion via enol/enolate mechanisms, and was prepared by *N*-acylation of serotonin hydrochloride (**7**) with lactam carboxylic acid **9**.[2] The remaining analogs introduced aromatic and heteroaromatic rings in place of the lactam, including *N*-(4-pyridinecarboxyl)serotonin (**3**) and *N*-benzoylserotonin (**4**)[3] as a more stable and sterically larger analog of *N*-acetylserotonin (NAS). *N*-Acylation of fluorinated pyridinecarboxylic acid isomers **12** and **13** provided the respective fluoropyridine analogs **5** and **6**. Isolated yields were modest, primarily due to care in chromatographic purification. These analogs were stable to prolonged storage and were typically kept in a -20 ^{o}C freezer in vials sparged with argon atmosphere.

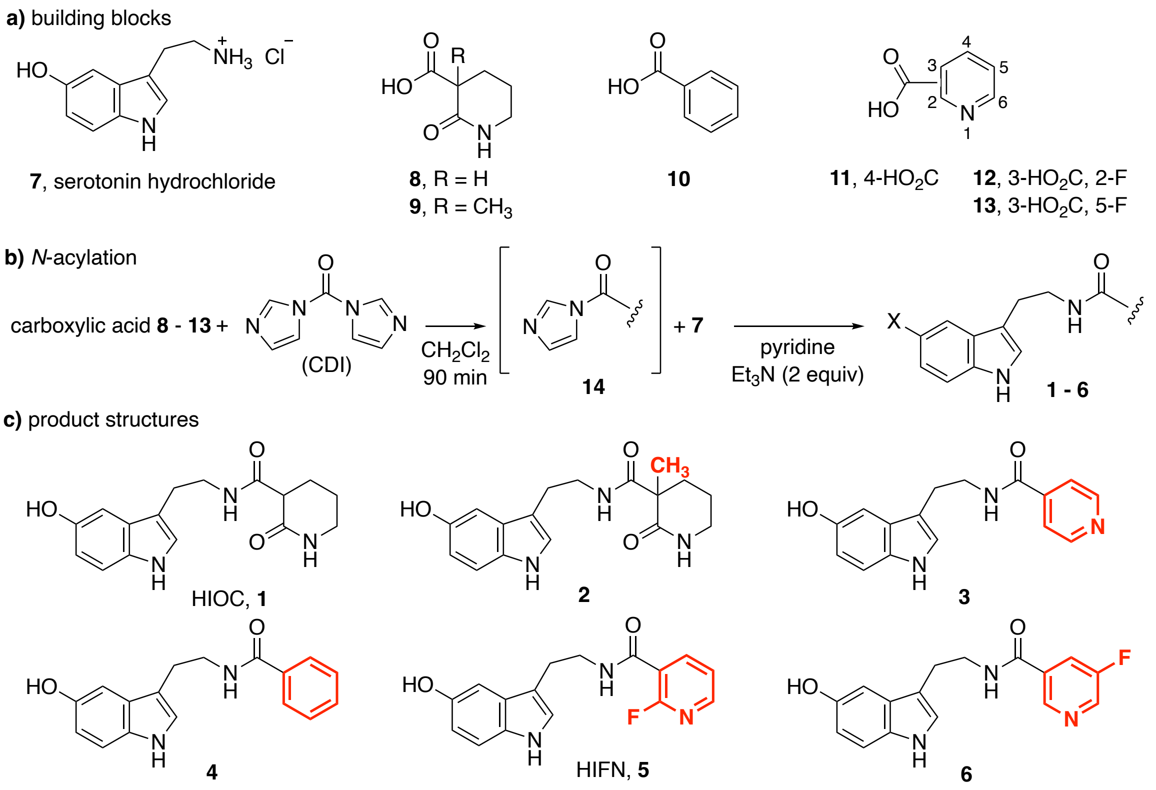

S1 Fig. Chemical synthesis of HIOC (**1**) and analogs **2** - **6**. Differences in analog structures from HIOC are highlighted in red.

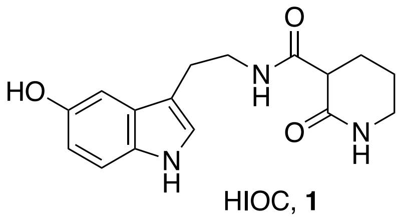

HIOC, **1**

N-(2-(5-Hydroxy-1H-indol-3-yl)ethyl)-2-oxopiperidine-3-carboxamide (HIOC, **1**)
SMILES: OC1=CC2=C([NH]C=C2CCNC(C(CCCN3)C3=O)=O)C=C1

To an oven-dried three-neck flask was added 2-oxopiperidine-3-carboxylic acid (**8**, 1.97 g, 13.8 mmol)[1] and 1,1'-carbonyldiimidazole (CDI, 2.26 g, 13.9 mmol, 1.01 equivalent) under argon atmosphere. Anhydrous dichloromethane (45 mL) was added, and the mixture was stirred for 1 hr. Serotonin hydrochloride (**7**, 2.93 g, 13.8 mmol, 1 equivalent) was added in one portion followed by anhydrous pyridine (45 mL). After 30 min the majority of serotonin hydrochloride was dissolved, however a small amount remained insoluble. Triethylamine (2.79 g, 27.6 mmol, 2 equivalent) was added, and the reaction mixture was stirred for 4 hrs at room temperature. After 4 hrs, the reaction mixture was analyzed by thin layer chromatography (TLC, ethyl acetate:methanol (9:1) eluent, stained with *p*-anisaldehyde). In addition to a new purple spot corresponding to product (HIOC, **1**), traces of serotonin were observed, which also stained purple. The reaction mixture was stirred for an additional 3 hrs, at which time TLC indicated that serotonin was consumed. The reaction mixture was concentrated by rotary evaporation, producing a viscous oil, which was dissolved in a minimal amount of ethyl acetate, to which silica gel was added to adsorb the crude product. After concentration by rotary evaporation to remove the ethyl acetate, the crude product mixture adsorbed on silica gel was dry loaded onto a chromatography column, and eluted via ethyl acetate : methanol gradient. Ethyl acetate : methanol (98 : 2, v/v) was used to elute the least polar material, after which concentration of methanol was increased to 5%, at which product (HIOC) began to elute. The concentration of methanol was increased to 7%, to elute the remainder of product (HIOC), as followed by TLC analysis. The combined fractions were concentrated by rotary evaporation to yield a sticky paste. Warm ether (10 mL) was added, the suspension was agitated with a spatula, and the ether was decanted. This process was repeated 4 times to produce a white solid. The white solid was then loaded on a fritted funnel and washed with additional warm ether (10 mL) and acetone (10 mL) to remove traces of imidazole impurities that had co-eluted with HIOC in the silica gel chromatography. Drying on frit produced HIOC (**1**, 2.7 g, 67% yield) as a white airy solid.

^{1}H-NMR (400 MHz, DMSO-d_6): 10.46 (s, 1H), 8.57 (s, 1H), 8.04 (dd, *J* = 5.6, 5.2 Hz, 1H), 7.64 (s, 1H), 7.09-7.07 (d, *J* = 8.4 Hz, 1H), 7.02-7.01 (d, *J* = 2.4 Hz, 1H), 6.80-6.79 (d, *J* = 2 Hz, 1H), 6.56-6.53 (dd, *J* = 6.4, 2.4 Hz, 1H), 3.28-3.21 (m, 2H), 3.11-3.08 (m, 2H), 3.05-3.02 (t, *J* = 6.2 Hz, 1H), 2.70-2.66 (t, *J* = 7.6 Hz, 1H), 1.90-1.85 (m, 1H), 1.81-1.72 (m, 3H), 1.56-1.53 (m, 1H).

^{13}C{^{1}H}-NMR (100 MHz, DMSO-d_6): 170.2, 168.8, 150.6, 131.2, 128.3, 123.6, 112.1, 111.6, 111.1, 102.6, 48.4, 41.8, 40.5, 25.6, 24.9, 20.9.

HRMS: $C_{16}H_{20}N_3O_3$ $[M+H]^+$ calculated 302.1499; found 302.1501

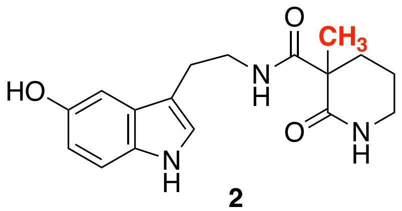

2

N-(2-(5-Hydroxy-1H-indol-3-yl)ethyl)-3-methyl-2-oxopiperidine-3-carboxamide (**2**):
SMILES: OC1=CC2=C([NH]C=C2CCNC(C(CCCN3)(C)C3=O)=O)C=C1

To an oven-dried three-neck flask was added 3-methyl-2-oxopiperidine-3-carboxylic acid (**9**, 330 mg, 2.10 mmol)[2] and 1,1'-carbonyldiimidazole (CDI, 337 mg, 2.08 mmol, 0.99 equivalent) under argon atmosphere. Anhydrous dichloromethane (6 mL) was added, and the mixture was stirred for 90 min. Serotonin hydrochloride (**7**, 446 mg, 2.09 mmol, 0.99 equivalent) was added in one portion followed by anhydrous pyridine (6 mL). After 15 min a small portion of serotonin hydrochloride remained insoluble whereupon triethylamine (424 mg, 4.19 mmol, 2 equivalents) was added forming a homogenous solution. The reaction mixture was stirred for overnight at room temperature. The reaction mixture was analyzed by thin layer chromatography (TLC, ethyl acetate:methanol (97:3) eluent, stained with *p*-anisaldehyde). A new purple spot corresponding to product **2** was observed. TLC indicated that serotonin hydrochloride was consumed. To the reaction mixture was added water (3 mL) and the mixture was transferred to a separatory funnel. After shaking, the heavier dichloromethane (organic) phase was separated from the lighter aqueous phase. In a separatory funnel, the organic phase was then washed with 5% (w/v) sodium bicarbonate solution (2 mL) with shaking and venting, and the process was repeated 3 times. The aqueous layer was removed, and the organic phase was then washed once with water (2 mL). The organic phase was washed with 5% acetic acid solution (2 mL) with shaking and venting, repeating the process 3 times. After separating the organic phase from the aqueous phase, the organic phase was then washed with water (2 mL). The organic layer was subsequently washed with 0.5 M aqueous HCl (2 mL) in the separatory funnel with shaking and venting, the process was repeated 3 times. After separating the aqueous phase, the organic phase was washed with 1M aqueous HCl (2 mL), and this process was repeated 3 times. Lastly the organic layer was washed with water (2 mL) followed by a brine wash (5 mL). The aqueous layer was removed, and the organic layer was dried using anhydrous sodium sulfate (Na_2SO_4). After filtration, the organic layer was concentrated by rotary evaporation, producing a viscous oil, which was dissolved in a minimal amount of ethyl acetate: methanol (97 : 3), to which silica gel was added to adsorb the crude product. After concentration by rotary evaporation to remove the ethyl acetate, the crude product mixture adsorbed on silica gel was dry loaded onto a chromatography column, and eluted via ethyl acetate : methanol gradient. Ethyl acetate : methanol (97 : 3, v/v) was used to elute the least polar material, after which concentration of methanol was increased to 5%, at which product (**2**) began to elute and was followed by TLC analysis (95 : 5, v/v). The combined fractions were concentrated by rotary evaporation to yield an airy white solid. The product **2** was produced in 25% yield (166 mg).

^{1}H-NMR (600 MHz, DMSO-d$_6$): 10.45 (s, 1H), 8.55 (s, 1H), 7.69 (s, 1H), 7.67-7.64 (t, *J* = 8.7, 1H), 7.09- 7.06 (d, *J* = 12.6 Hz, 1H), 6.97 (d, *J* = 3.6 Hz, 1H), 6.82-6.81 (d, *J* = 3.6

Hz, 1H), 6.56-6.53 (dd, J = 3.6 Hz, 1H), 3.26-3.23 (m, 2H), 3.09-3.06 (t, J = 4.8 Hz, 2H), 2.69-2.65 (t, J = 10.8 Hz, 2H), 2.26-2.21 (m, 1H), 1.60-1.41 (m, 3H), 1.23 (s, 3H).

$^{13}C\{^{1}H\}$-NMR (150 MHz, DMSO-d$_6$): 173.2, 172.2, 150.6, 131.2, 128.3, 123.6, 112.0, 111.7, 111.0, 102.7, 49.2, 42.0, 40.5, 31.8, 25.6, 25.1, 19.9.

HRMS: $C_{17}H_{22}N_3O_3$ $[M+H]^+$ calculated 316.1656; found 316.1658

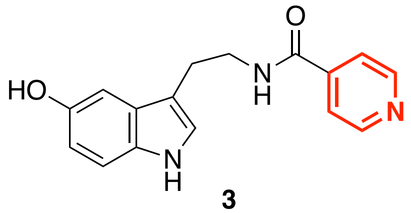

3

N-(2-(5-Hydroxy-1H-indol-3-yl)ethyl)isonicotinamide (**3**):
SMILES: OC1=CC2=C([NH]C=C2CCNC(C3=CC=NC=C3)=O)C=C1

To an oven-dried three-neck flask was added isonicotinic acid (**11**, 250 mg, 2.03 mmol) and 1,1'-carbonyldiimidazole (CDI, 326 mg, 2.01 mmol, 0.99 equivalent) under argon atmosphere. Anhydrous dichloromethane (7 mL) was added, and the mixture was stirred for 30 min. Serotonin hydrochloride (**7**, 427 mg, 2.01 mmol, 0.99 equivalent) was added in one portion followed by anhydrous pyridine (7 mL). After 15 min a small portion of serotonin hydrochloride remained insoluble whereupon triethylamine (407 mg, 4.02 mmol, 2 equivalents) was added forming a homogenous solution. The reaction mixture was stirred for 8 hrs at room temperature. The reaction mixture was analyzed by thin layer chromatography (TLC, ethyl acetate:methanol (90:10) eluent, stained with *p*-anisaldehyde). A new purple spot corresponding to product (**3**) was observed. TLC indicated that serotonin hydrochloride was consumed. To the reaction mixture was added water (3 mL) and the mixture was transferred to a separatory funnel. After shaking, the heavier dichloromethane (organic) phase was separated from the lighter aqueous phase. In a separatory funnel, the organic phase was then washed with 5% (w/v) sodium bicarbonate solution (2 mL) with shaking and venting, and the process was repeated 3 times. The aqueous layer was removed, and the organic phase was then washed once with water (2 mL). The organic phase was washed with 5% acetic acid solution (2 mL) with shaking and venting, repeating the process 3 times. After separating the organic phase from the aqueous phase, the organic phase was then washed with water (2 mL). The organic layer was subsequently washed with 0.5 M aqueous HCl (2 mL) in the separatory funnel with shaking and venting, the process was repeated 3 times. After separating the aqueous phase, the organic phase was washed with 1M aqueous HCl (2 mL), and this process was repeated 3 times. Lastly the organic layer was washed with water (2 mL) followed by a brine wash (5 mL). The aqueous layer was removed, and the organic layer was dried using anhydrous sodium sulfate (Na_2SO_4). After filtration, the organic layer was concentrated by rotary evaporation, producing a viscous oil, which was dissolved in a minimal amount of ethyl acetate: methanol (90 : 10), to which silica gel was added to adsorb the crude product. After concentration by rotary evaporation to remove the ethyl acetate, the crude product mixture adsorbed on silica gel was dry loaded onto a chromatography column, and eluted via ethyl acetate : methanol (95:5). Fractions monitored by TLC analysis (95 : 5, v/v). The combined

fractions were concentrated by rotary evaporation to yield a solid. The product **3** was produced in 36% yield (204 mg).

^{1}H-NMR (600 MHz, DMSO-d$_6$): 10.47 (s, 1H), 8.87-8.85 (t, J = 5.4 Hz, 1H), 8.69-8.68 (d, J = 5.4 2H), 8.57 (s, 1H), 7.72-7.71 (d, J = 6 Hz, 2H), 7.09-7.08 (d, J = 9 Hz, 1H), 7.04 (s, 1H), 6.84 (s, 1H) 6.56-6.55 (dd, J = 8.6, 2.3 Hz, 1H), 3.49-3.46 (q, J = 7.2 Hz, 2H), 2.84-2.81 (t, J = 7.5 Hz, 2H).

HRMS: $C_{16}H_{16}N_3O_2$ $[M+H]^+$ calculated 282.1237; found 282.1233

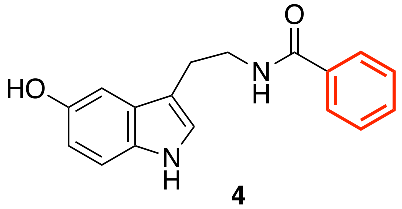

4

N-(2-(5-Hydroxy-1H-indol-3-yl)ethyl)benzamide (**4**):[3]
SMILES: OC1=CC2=C([NH]C=C2CCNC(C3=CC=CC=C3)=O)C=C1

To an oven-dried three-neck flask was added benzoic acid (**10**, 350 mg, 2.87 mmol) and 1,1'-carbonyldiimidazole (CDI, 460 mg, 2.84 mmol, 0.99 equivalent) under argon atmosphere. Anhydrous dichloromethane (9.5 mL) was added, and the mixture was stirred for 30 min. Serotonin hydrochloride (**7**, 603 mg, 13.8 mmol, 1 equivalent) was added in one portion followed by anhydrous pyridine (9.5 mL). After 30 min the majority of serotonin hydrochloride was dissolved, however a small amount remained insoluble. Triethylamine (581 mg, 5.74 mmol, 2 equivalent) was added, and the reaction mixture was stirred for 8 hrs at room temperature. After 8 hrs, the reaction mixture was analyzed by thin layer chromatography (TLC, hexanes:ethyl acetate) (1:1) eluent, stained with *p*-anisaldehyde). A new purple spot corresponding to product (**4**) was observed. The reaction mixture was concentrated by rotary evaporation, producing a viscous oil, which was dissolved in a minimal amount of ethyl acetate, to which silica gel was added to adsorb the crude product. After concentration by rotary evaporation to remove the ethyl acetate, the crude product mixture adsorbed on silica gel was dry loaded onto a chromatography column and eluted via hexanes: ethyl acetate (1:1, v/v) as followed by TLC analysis. The combined fractions were concentrated by rotary evaporation to yield a solid product **4** in 17% yield (135 mg).

The synthesis of compound **4** was previously reported by a different procedure. Our ^{1}H NMR spectrum closely correlates to their published data.[3]

^{1}H-NMR (600 MHz, DMSO-d$_6$): 10.47 (s, 1H), 8.59 (s, 1H), 8.58-8.56 (t, J = 5.7 Hz, 1H), 7.83-7.82 (d, J = 7.4 Hz, 2H), 7.50-7.47 (t, J = 7.2 Hz, 1H), 7.44-7.42 (t, J = 7.8 Hz, 2H), 7.12-7.09 (d, J = 7.8 Hz, 1H), 7.05-7.04 (d, J = 1.8 Hz, 1H), 6.87 (d, J = 1.8 Hz, 1H), 6.58-6.56 (dd, J = 8.6, 2.4 Hz, 1H), 3.50-3.47 (q, J = 6.6 Hz, 2H), 2.84-2.82 (t, J = 7.5 Hz, 2H).

^{13}C{^{1}H}-NMR (150 MHz, DMSO-d$_6$): 166.5, 150.6, 135.2, 131.5, 131.2, 128.7, 128.4, 127.6, 123.5, 112.1, 111.7, 111.3, 102.7, 40.5, 25.8.

HRMS: $C_{17}H_{17}N_2O_2$ $[M+H]^+$ calculated 281.1285; found 281.1287

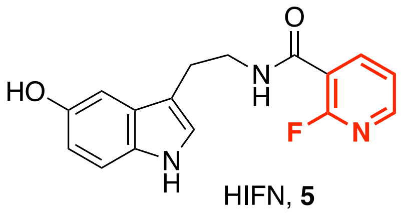

HIFN, **5**

2-Fluoro-*N*-(2-(5-hydroxy-1H-indol-3-yl)ethyl)nicotinamide (HIFN, **5**):
SMILES: OC1=CC2=C([NH]C=C2CCNC(C3=C(F)N=CC=C3)=O)C=C1

To an oven-dried flask was added 2-fluoronicotinic acid (**12**, 1.00 g, 7.09 mmol) and 1,1'-carbonyldiimidazole (CDI, 1.15 g, 7.09 mmol, 1 equivalent) under argon atmosphere. Anhydrous dichloromethane (DCM, 20 mL) was added, and the mixture was stirred for 30 min. Serotonin hydrochloride (**7**, 1.51 g, 7.09 mmol, 1 equivalent) was added in one portion followed by anhydrous pyridine (20 mL). After 5 min, 80% dissolution of serotonin hydrochloride was observed whereupon triethylamine (TEA, 1.43 g, 14.2 mmol, 2 equivalents) was added. The reaction mixture was stirred for 12 h at room temperature. To the reaction mixture was added water (equal volume to that of pyridine - 20 mL) and the mixture was transferred to a separatory funnel. After shaking, the heavier dichloromethane (organic) phase was separated from the lighter aqueous phase. In a separatory funnel, the organic phase was then washed with 5% (w/v) sodium bicarbonate solution (10 mL) with shaking and venting, and the process was repeated 3 times. The aqueous layer was removed, and the organic phase was then washed once with water (10 mL). The organic phase was washed with 5% acetic acid solution (10 mL) with shaking and venting, repeating the process 3 times. After separating the organic phase from the aqueous phase, the organic phase was then washed with water (10 mL). The organic layer was subsequently washed with 0.5 M aqueous HCl (10 mL) in the separatory funnel with shaking and venting, the process was repeated 3 times. After separating the aqueous phase, the organic phase was washed with 1M aqueous HCl (10 mL), and this process was repeated 3 times. Lastly the organic layer was washed with water (10 mL). The aqueous layer was removed, and the organic layer was concentrated by rotary evaporation, producing a brown solid. Room temperature methanol was added to the solid, the methanol became a brownish color and insoluble powder remained. Agitation via scratching with a spatula caused more solid to precipitate out, the heterogenous mixture was heated and filtered while hot yielding a light tan-off white solid. The mother liquor was concentrated by rotary evaporation producing a darker brown solid. Cold methanol was added to the solid creating a heterogenous mixture (a brown solution and a tan powder). Once again agitation via scratching with a spatula induced greater precipitation. The heterogenous solution was heated and the insoluble material filtered while hot collecting additional tan solid. The mother liquor was concentrated via rotary evaporation a second time producing a richer brown solid addition of a small amount of methanol produced a small amount of insoluble material which upon heating generated a homogenous solution which upon standing cooled to room temperature and was observed for crystallization. The HIFN product **5** was produced in 42% yield (860 mg).

An alternative method to chromatography for purification was created using warm methanol to crystallize **5** from the crude oil. By ^{1}H NMR spectroscopic analysis, solutions

derived from crystalline **5** are identical to previous samples purified by silica gel chromatography, and their biological activity was verified in vivo. This chemical synthesis result was consistently achieved when using 2-fluoronicotinic acid (**12**) and CDI sourced from Sigma Aldrich, however materials obtained from other sources rendered the purification protocol inconsistent. This variation presumably arises from impurities in the source material, although the nature of those impurities was not obvious. In addition to the impurities, the presence of pyridine in the crude product mixture increases the solubility of the crude product, hindering crystallization.

In our modified protocol we sought to address both issues. The reaction setup, molar equivalency, and concentrations remained unchanged; modifications were incorporated during processing of the reaction. Upon reaction completion, the amount of water added to the reaction was increased to equal the volume to that of pyridine and the mixture was stirred for 3 minutes. Additional modifications included increasing the volume of washing solutions to half the volume of pyridine per wash. At the conclusion of the successive washes, the organic layer was removed and concentrated to yield a brown solid. Room temperature methanol was added to the crude solid, and agitation via scratching with a spatula induced precipitation. Interestingly the methanol retained the brownish color and not the insoluble powder. Previous experiments have identified methanol as a suitable recrystallization agent for **5**, so the insoluble product at this point was presumed to be **5**. To ensure only insoluble material was collected, the heterogenous mixture was heated and filtered while hot, yielding a light tan-off white solid. The mother liquor was concentrated by rotary evaporation producing a darker brown solid. Cold methanol was added to the solid creating a heterogenous mixture (a brown solution and a tan powder). Once again agitation via scratching with a spatula induced greater precipitation. The heterogenous solution was heated and the insoluble material filtered while hot, collecting additional tan solid. The mother liquor was concentrated via rotary evaporation for a second time, producing a richer brown solid. Addition of a small amount of methanol produced a small amount of insoluble material, which upon heating generated a homogenous solution, which upon standing cooled to room temperature. The modified protocol produced a 42% yield of HIFN (**5**), consistent to what we observed with our previous crystallization method and successfully avoids the use of column chromatography.

^{1}H-NMR (600 MHz, DMSO-d_6): 10.52 (br d, J = 2.4 Hz, 1H), 8.61 (br s, 1H), 8.61 (app br t, J = 5.2 Hz, 1H), 8.35 (ddd, J = 1.0, 2.0, 4.8 Hz, 1H), 8.15 (ddd, J = 2.0, 7.4, 9.5 Hz, 1H), 7.46 (ddd, J = 2.0, 4.8, 7.1 Hz, 1H), 7.13 (br d, J = 8.6 Hz, 1H), 7.09 (d, J = 2.4 Hz, 1H), 6.88 (d, J = 2.3 Hz, 1H), 6.60 (dd, J = 2.3, 8.6 Hz, 1H), 3.50 (br td, J = 7.0, 7.6 Hz, 2H), 2.85 (t, J = 7.5 Hz, 2H).

^{13}C{^{1}H}-NMR (150 MHz, DMSO-d_6): 162.9 (d, J_{CF} = 5.5 Hz), 159.6 (d, J_{CF} = 239 Hz), 150.7, 149.6 (d, J_{CF} = 15.0 Hz), 141.9 (d, J_{CF} = 3.6 Hz), 131.3, 128.3, 123.7, 122.7 (d, J_{CF} = 4.3 Hz), 119.4 (d, J_{CF} = 30.2 Hz), 112.2, 111.7, 111.0, 102.7, (40 ppm obscured by solvent), 25.8.

HRMS: $C_{16}H_{15}N_3O_2F$ $[M+H]^+$ calculated 300.1143; found 300.1140

S1 Table. Tabulated ^{13}C and ^{1}H NMR data for HIFN (**5**)

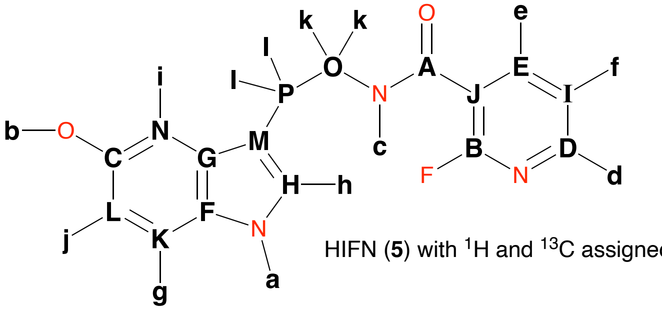

^{13}C ppm, coupling	correlated ^{1}H ppm, coupling	^{1}H-^{1}H COSY proton letter	^{1}H-^{13}C HMBC carbon letter
(indole N-H)	**a**, 10.52, br d, $J = 2.4$ Hz	**h**	**G**, **H**, **M**
(phenol O-H)	**b**, 8.61, br s	--	**L**, **N**
(amide N-H)	**c**, 8.61, app br t, $J = 5.2$ Hz	**k$_2$**	**A**
**A**, 162.9, d, $^3J_{CF}$ 5.5 Hz	--	--	--
**B**, 159.6, d, $^1J_{CF}$ 239 Hz	--	--	--
**C**, 150.7	--	--	--
**D**, 149.6, d, $^3J_{CF}$ 15.0 Hz	**d**, 8.35, ddd, $J = 1.0, 2.0, 4.8$ Hz	**f**	**B**, **E**, **I**
**E**, 141.9, d, $^3J_{CF}$ 3.6 Hz	**e**, 8.15, ddd, $J = 2.0, 7.4, 9.5$ Hz	**f**	**A**, **B**, **D**
**F**, 131.3	--	--	
**G**, 128.3	--	--	
**H**, 123.7	**h**, 7.09, d, $J = 2.4$ Hz	**a**	**F**, **M**, **P**
**I**, 122.7, d, $^4J_{CF}$ 4.3 Hz	**f**, 7.46, ddd, $J = 2.0, 4.8, 7.1$ Hz	**d**, **e**	**D**, **J**
**J**, 119.4, d, $^2J_{CF}$ 30.2 Hz	--	--	
**K**, 112.2	**g**, 7.13, br d, $J = 8.6$ Hz	**j**	**C**, **G**, **N**
**L**, 111.7	**j**, 6.60, dd, $J = 2.3, 8.6$	**g**, **i**	**F**, **N**
**M**, 111.0	--	--	--
**N**, 102.7	**i**, 6.88, d, $J = 2.3$ Hz	**j**	**C**, **F**, **L**
**O**, 40.5*	**k$_2$**, 3.50, br td, $J = 7.0, 7.6$ Hz	**c**, **l$_2$**	**A**, **M**, **P**
**P**, 25.8	**l$_2$**, 2.85, t, $J = 7.5$ Hz	**k$_2$**	**G**, **H**, **M**, **O**

* resonance obscured by DMSO-d_6, but identified by HSQC and HMBC correlations

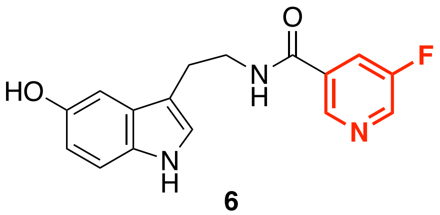

6

5-Fluoro-*N*-(2-(5-hydroxy-1H-indol-3-yl)ethyl)nicotinamide (**6**):
SMILES: OC1=CC2=C([NH]C=C2CCNC(C3=CN=CC(F)=C3)=O)C=C1

To an oven-dried three-neck flask was added 5-fluoro-3-pyridine carboxylic acid (**13**, 300 mg, 2.13 mmol) and 1,1'-carbonyldiimidazole (CDI, 341 mg, 2.10 mmol, 0.99 equivalent) under argon atmosphere. Anhydrous dichloromethane (6.5 mL) was added, and the mixture was stirred for 30 min. Serotonin hydrochloride (**7**, 452 mg, 2.10 mmol, 0.99 equivalent) was added in one portion followed by anhydrous pyridine (6.5 mL). After 10 mins complete dissolution of serotonin hydrochloride was observed whereupon triethylamine (430 mg, 4.25 mmol, 2 equivalents) was added. The reaction mixture was stirred for 12 hrs at room temperature. The reaction mixture was analyzed by thin layer chromatography (TLC, hexanes:ethyl acetate (40:60) eluent, stained with *p*-anisaldehyde). A new purple spot corresponding to product (**6**) was observed. TLC indicated that serotonin hydrochloride was consumed. To the reaction mixture was added water (3 mL) and the mixture was transferred to a separatory funnel. After shaking, the heavier dichloromethane (organic) phase was separated from the lighter aqueous phase. In a separatory funnel, the organic phase was then washed with 5% (w/v) sodium bicarbonate solution (2 mL) with shaking and venting, and the process was repeated 3 times. The aqueous layer was removed, and the organic phase was then washed once with water (2 mL). The organic phase was washed with 5% acetic acid solution (2 mL) with shaking and venting, repeating the process 3 times. After separating the organic phase from the aqueous phase, the organic phase was then washed with water (2 mL). The organic layer was subsequently washed with 0.5 M aqueous HCl (2 mL) in the separatory funnel with shaking and venting, the process was repeated 3 times. After separating the aqueous phase, the organic phase was washed with 1M aqueous HCl (2 mL), and this process was repeated 3 times. Lastly the organic layer was washed with water (2 mL) followed by a brine wash (5 mL). The aqueous layer was removed, upon standing a precipitate begun to form in the organic layer which was redissolved by addition of 5 mL of methanol and the organic layer was dried using anhydrous sodium sulfate (Na_2SO_4). After filtration, the organic layer was concentrated by rotary evaporation, producing a viscous oil, which was dissolved in a minimal amount of ethyl acetate, to which silica gel was added to adsorb the crude product. After concentration by rotary evaporation to remove the ethyl acetate, the crude product mixture adsorbed on silica gel was dry loaded onto a chromatography column, and eluted via hexanes: ethyl acetate gradient. Hexanes : ethyl acetate (40 : 60, v/v) was used to elute the least polar material, after which concentration of ethyl acetate was increased to 70%, at which remaining non-polar material was eluted, product (**6**) began to elute at 80% ethyl acetate and was followed by TLC analysis (40 :60, hexane:ethyl acetate, v/v). The combined fractions were concentrated by rotary evaporation to yield a solid. The product **6** was produced in 25% yield (159 mg).

^{1}H-NMR (600 MHz, DMSO-d$_6$): 10.47 (s, 1H), 8.85 (br s, 2H), 8.70-8.69 (d, *J* = 2.4 Hz, 1H), 8.58 (s, 1H), 8.03-8.02 (d, *J* = 9.6 Hz, 1H), 7.10-7.09 (d, *J* = 8.4 Hz, 1H), 7.05 (s, 1H), 6.84 (s, 1H) 6.57-6.56 (d, *J* = 7.8 Hz, 1H), 3.51-3.48 (q, *J* = 6.6 Hz, 2H), 2.85-2.83 (t, *J* = 7.5 Hz, 2H).

HRMS: $C_{16}H_{15}N_3O_2F$ $[M+H]^+$ calculated 300.1143; found 300.1140

Configurational stability of HIOC (1) by deuterium exchange

To test the configurational stability of the stereogenic carbon of HIOC, we assessed the extent of deuterium incorporation with a deuterated phosphate buffer, in a pD range of 7.3 - 8.1, which corresponded to a pH range of 6.9 - 7.7 approximating physiological conditions. For a solution of HIOC (**1**) in d^6-DMSO at 37 ºC with 0.1 M phosphate buffer, we observed rapid disappearance of the diagnostic methine proton (3.03 ppm), at all pD tested. This is evidence for facile protium-deuterium exchange via an achiral enol or enolate intermediate (**Fig S2**). Notably, the methine proton was unchanged simply with D_2O in the absence of buffer. This experiment demonstrated that an enantioselective synthesis of HIOC was impractical.

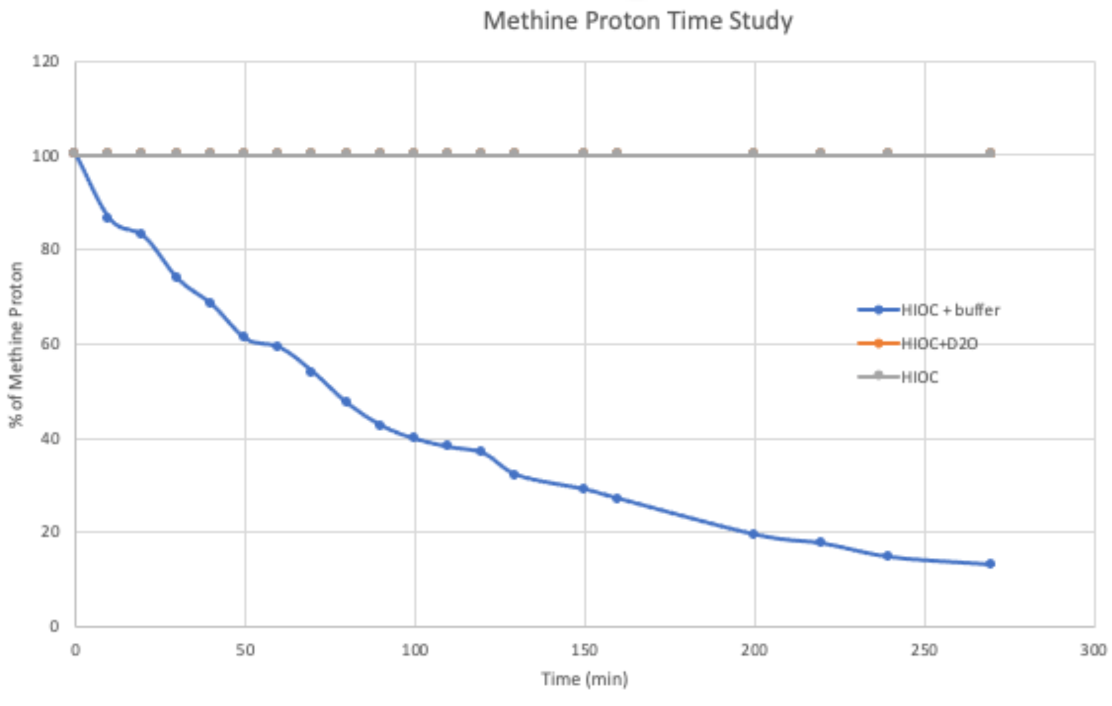

S2 Fig. Time course of protium-deuterium exchange with HIOC (**1**) at methine carbon, at pD 7.8

Crystal structure data

N-(2-(5-Hydroxy-1H-indol-3-yl)ethyl)-2-oxopiperidine-3-carboxamide (HIOC, **1**)

We obtained a X-ray crystal structure from a racemic batch of HIOC (**1**, **S1 Fig**). This revealed that crystals of HIOC contained both (*R*)- and (*S*)-enantiomers, ruling out spontaneous resolution (**S3 Fig**).[4] However, the crystal structure exhibited a conformation with orthogonal orientation of the two carbonyls (relative torsion angle 66°), with 2° amide torsion angle of 7°, so that only one carbonyl was suitably aligned with the C-H bond at the stereogenic carbon for enolization to the achiral enol/enolate. Notably, the ring carbonyl was not in proximity with the secondary amide hydrogen, precluding intramolecular hydrogen bonding, also consistent with ^{1}H NMR chemical shift (N-H at 8.04 ppm, doublet of doublets, 5.2, 5.6 Hz).

Experimental. Single colourless needle crystals of HIOC (**1**) were chosen from the sample as supplied. A suitable crystal with dimensions 0.28 × 0.12 × 0.08 mm^3 was selected and mounted on a loop with paratone on a XtaLAB Synergy-S diffractometer. The crystal was kept at a steady T = 101(2) K during data collection The structure was solved with the ShelXT solution program using dual methods and by using Olex2 as the graphical interface.[5,6] The model was refined with olex2.refine 1.3-dev using full matrix least squares minimisation on $\boldsymbol{\Phi^2}$.[7]

Crystal Data. $C_{16}H_{19}N_3O_3$, M_r = 301.348, monoclinic, $P2_1/n$ (No. 14), a = 4.85886(8) Å, b = 10.58127(17) Å, c = 28.4331(5) Å, β = 92.2265(15)°, $\alpha = \gamma$ = 90°, V = 1460.73(4) Å^3, T = 101(2) K, Z = 4, Z' = 1, μ(Cu K_α) = 0.789 mm^{-1}, 16272 reflections measured, 2780 unique (R_{int} = 0.0360) which were used in all calculations. The final wR_2 was 0.0716 (all data) and R_1 was 0.0311 ($I \geq 2\ \sigma(I)$).

CCDC deposition number 2366626

**Compound**	**HIOC (1)**
Formula	$C_{16}H_{19}N_3O_3$
$D_{calc.}$/ g cm^{-3}	1.370
μ/mm^{-1}	0.789
Formula Weight	301.348
Color	colorless
Shape	needle
Size/mm^3	0.28×0.12×0.08
T/K	101(2)
Crystal System	monoclinic
Space Group	$P2_1/n$
a/Å	4.85886(8)
b/Å	10.58127(17)
c/Å	28.4331(5)
α/°	90
β/°	92.2265(15)
γ/°	90
V/Å^3	1460.73(4)
Z	4
Z'	1
Wavelength/Å	1.54184
Radiation type	Cu K_α
Θ_{min}/°	3.11
Θ_{max}/°	72.77
Measured Refl's.	16272
Indep't Refl's	2780
Refl's I≥2 σ(I)	2576
R_{int}	0.0360
Parameters	371
Restraints	54
Largest Peak	0.1886
Deepest Hole	-0.1920
GooF	1.1077
wR_2 (all data)	0.0716
wR_2	0.0705
R_1 (all data)	0.0337
R_1	0.0311

Structure Quality Indicators

**Reflections:**	d min (Cu) 0.81	I/σ(I) 47.6	Rint 3.59%	complete 100% (IUCr) 99%
**Refinement:**	Shift 0.000	Max Peak 0.2	Min Peak -0.2	GooF 1.108

A colorless needle-shaped crystal of HIOC (**1**) with dimensions 0.28 × 0.12 × 0.08 mm^3 was mounted on a loop with paratone. Data were collected using a XtaLAB Synergy, Dualflex, HyPix diffractometer equipped with an Oxford Cryosystems low-temperature device operating at T = 101(2) K.

Data were measured using w scans using Cu K_α radiation. The diffraction pattern was indexed and the total number of runs and images was based on the strategy calculation from the program CrysAlisPro (Rigaku, V1.171.40.84a, 2020). The maximum resolution that was achieved was $\Theta = 72.77^\circ$ (0.81 Å).

The unit cell was refined using CrysAlisPro (Rigaku, V1.171.40.84a, 2020) on 7679 reflections, 47% of the observed reflections.

Data reduction, scaling and absorption corrections were performed using CrysAlisPro (Rigaku, V1.171.40.84a, 2020). The final completeness is 99.43 % out to 72.77° in Θ. A numerical absorption correction based on Gaussian integration over a multifaceted crystal model was performed using CrysAlisPro 1.171.40.84a (Rigaku Oxford Diffraction, 2020). An empirical absorption correction using spherical harmonics, implemented in SCALE3 ABSPACK scaling algorithm.. The absorption coefficient μ of this material is 0.789 mm^{-1} at this wavelength (λ = 1.54184Å) and the minimum and maximum transmissions are 0.643 and 1.000.

The structure was solved and the space group $P2_1/n$ (# 14) determined by the ShelXT 2018/2 structure solution program using dual methods and refined by full matrix least squares minimisation on $\boldsymbol{\Phi}^2$ using version of olex2.refine 1.3-dev.[7,8] All atoms including hydrogens were refined anisotropically. Hydrogen atom positions were located and freely refined.

_refine_special_details: Refinement using NoSpherA2, an implementation of NOn-SPHERical Atom-form-factors in Olex2.[9] 2020NoSpherA2 implementation of HAR makes use of tailor-made aspherical atomic form factors calculated on-the-fly from a Hirshfeld-partitioned electron density (ED) - not from spherical-atom form factors.The ED is calculated from a gaussian basis set single determinant SCF wavefunction - either Hartree-Fock or DFT using selected functionals - for a fragment of the crystal. This fregment can be embedded in an electrostatic crystal field by employing cluster charges. The following options were used: SOFTWARE: 'Please Select' PARTITIONING: NoSpherA2 INT ACCURACY: Normal METHOD: B3LYP BASIS SET: def2-SVP CHARGE: 0 MULTIPLICITY: 0 DATE: 2020-09-04_16-12-02

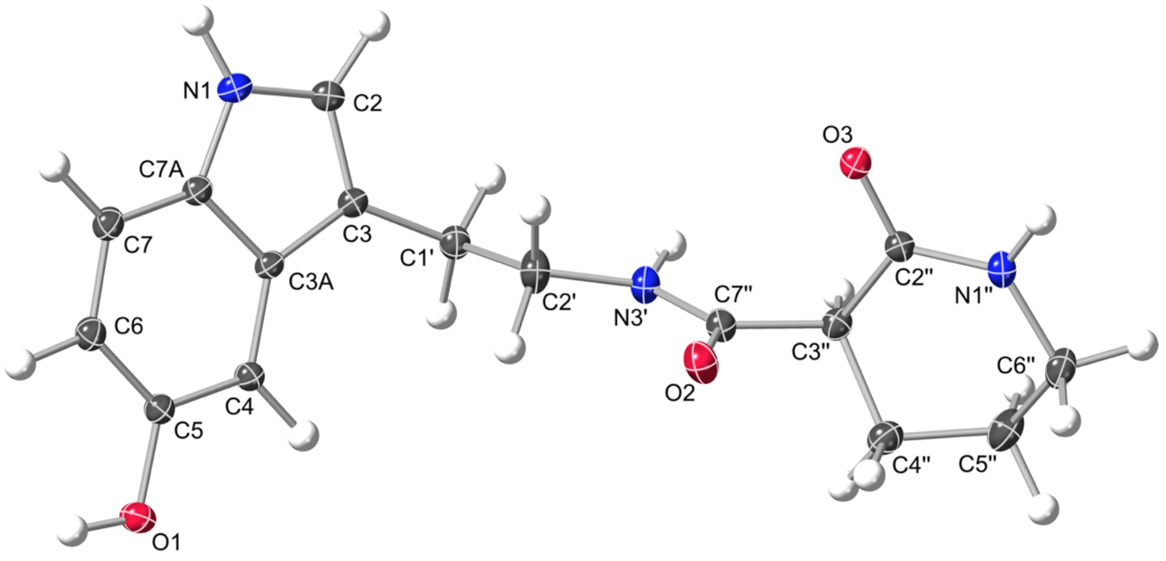

S3 Fig: Thermal ellipsoid representation of the asymmetric unit of HIOC (**1**). There is one molecule of the target compound in the asymmetric unit, which is represented by the reported sum formula. In other words: Z is 4 and Z' is 1. The chiral centers have both R and S-configuration in this crystal.

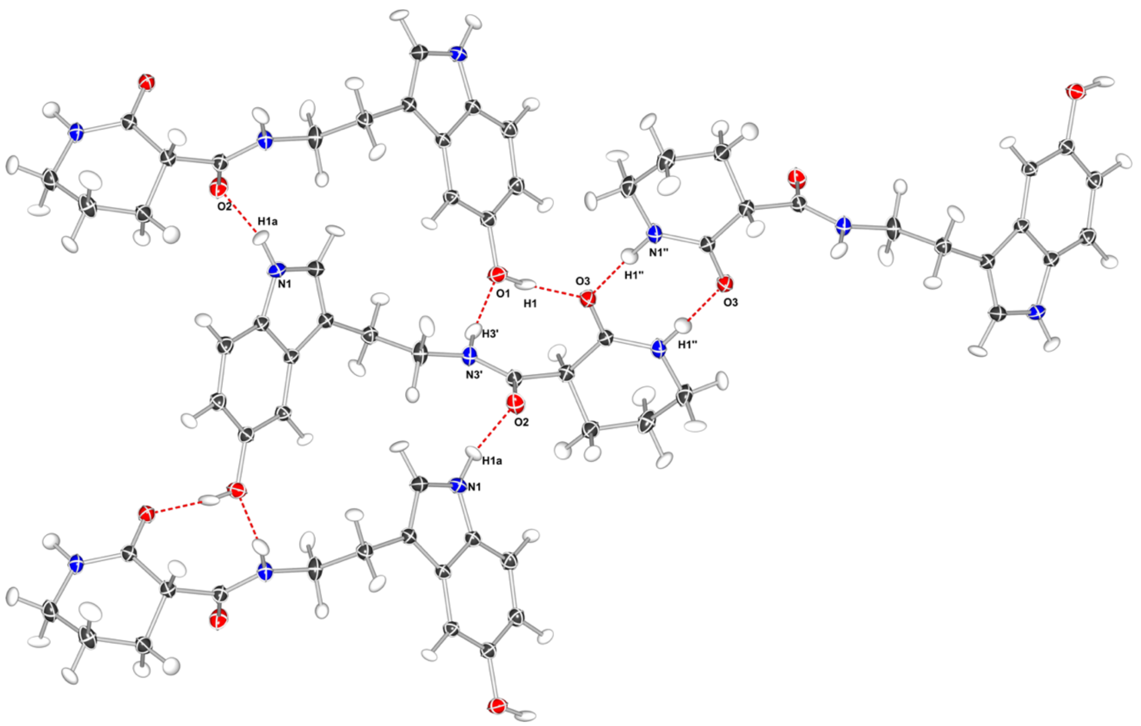

S4 Fig: Plot of a portion of the hydrogen bonding network in the crystal structure of HIOC (**1**).

2-Fluoro-*N*-(2-(5-hydroxy-1H-indol-3-yl)ethyl)nicotinamide (HIFN, **5**):

The X-ray crystal structure of analog **5** exhibited a distinct intramolecular H-F close contact between the 2° amide N-H and the fluorine substituent on the pyridine ring (**S5-S6 Fig**).[10] Furthermore, this part of the structure was nearly planar (relative torsion angle 174°), with the 2° amide torsion angle of 3°. This places the electronegative fluorine and nitrogen atoms of analog **5** in a similar but not identical conformation as the electronegative oxygen and nitrogen atoms of HIOC (**1**), leaving intact the other structural features of *N*-acetylserotonin.

Experimental. Single colorless prism-shaped crystals of **5** were chosen from the sample as supplied. A suitable crystal with dimensions 0.40 × 0.37 × 0.24 mm^3 was selected and mounted on a loop with paratone on a Synergy-S diffractometer. The crystal was kept at a steady T = 105(7) K during data collection. The structure was solved with the **ShelXT** 2018/2 solution program using dual-space methods and by using **Olex2** 1.5-alpha as the graphical interface.[6,8] The model was refined with **olex2.refine** 1.5-alpha using full matrix least squares minimisation on $\boldsymbol{\Phi}^2$.[7]

Crystal Data. $C_{16}H_{14}FN_3O_2$, M_r = 299.307, monoclinic, $P2_1/c$ (No. 14), a = 9.5199(4) Å, b = 10.2207(4) Å, c = 14.9286(6) Å, β = 107.353(4)°, $\alpha = \gamma$ = 90°, V = 1386.43(10) Å^3, T = 105(7) K, Z = 4, Z' = 1, μ(Cu K_α) = 0.886, 16922 reflections measured, 2722 unique (R_{int} = 0.0493) which were used in all calculations. The final wR_2 was 0.0398 (all data) and R_1 was 0.0187 (I≥2 s(I)).

CCDC deposition number 2366286

**Compound**	**HIFN (5)**
Formula	$C_{16}H_{14}FN_3O_2$
$D_{calc.}$/ g cm^{-3}	1.434
μ/mm^{-1}	0.886
Formula Weight	299.307
Color	colorless
Shape	prism-shaped
Size/mm^3	0.40×0.37×0.24
T/K	105(7)
Crystal System	monoclinic
Space Group	$P2_1/c$
a/Å	9.5199(4)
b/Å	10.2207(4)
c/Å	14.9286(6)
α/°	90
β/°	107.353(4)
γ/°	90
V/Å^3	1386.43(10)
Z	4
Z'	1
Wavelength/Å	1.54184
Radiation type	Cu K_α
Θ_{min}/°	4.87
Θ_{max}/°	73.06
Measured Refl's.	16922
Indep't Refl's	2722
Refl's I≥2 σ(I)	2547
R_{int}	0.0493
Parameters	401
Restraints	9
Largest Peak	0.1260
Deepest Hole	-0.1151
GooF	1.1476
wR_2 (all data)	0.0398
wR_2	0.0389
R_1 (all data)	0.0214
R_1	0.0187

Structure Quality Indicators

**Reflections:**	d min (Cu\a) 2Θ=146.5° 0.81	I/σ(I) 42.8	Rint 4.93%	Full 135.4° 98% to 146.5° 99.9
**Refinement:**	Shift 0.000	Max Peak 0.1	Min Peak -0.1	GooF 1.148

A colorless prism-shaped crystal of HIFN (**5**) with dimensions 0.40 × 0.37 × 0.24 mm^3 was mounted on a loop with paratone. Data were collected using a Synergy-S diffractometer equipped with an Oxford Cryosystems low-temperature device operating at T = 105(7) K.

Data were measured using ω scans with Cu K_α radiation. The diffraction pattern was indexed and the total number of runs and images was based on the strategy calculation from the program CrysAlisPro 1.171.41.98a (Rigaku OD, 2021). The maximum resolution that was achieved was Q = 73.06° (0.81 Å).

The unit cell was refined using CrysAlisPro 1.171.41.98a (Rigaku OD, 2021) on 3306 reflections, 20% of the observed reflections.

Data reduction, scaling and absorption corrections were performed using CrysAlisPro 1.171.41.98a (Rigaku OD, 2021). The final completeness is 99.92 % out to 73.06° in Θ. A Gaussian absorption correction using a multifaceted crystal model was performed using CrysAlisPro 1.171.41.108a (Rigaku Oxford Diffraction, 2021). An empirical absorption correction using spherical harmonics, implemented in SCALE3 ABSPACK scaling algorithm was also performed. The absorption coefficient μ of this material is 0.886 mm^{-1} at this wavelength (λ = 1.54184Å) and the minimum and maximum transmissions are 0.244 and 1.000.

The structure was solved and the space group $P2_1/c$ (# 14) determined by the **ShelXT** 2018/2 structure solution program using dual-space methods and refined by full matrix least squares minimisation on $\boldsymbol{\Phi^2}$ using version of **olex2.refine** 1.5-alpha.[7,8] All atoms, even hydrogen atoms, were refined anisotropically. Hydrogen atom positions were located from the electron densities and freely refined using Hirshfeld scattering factors. Refinement was by using NoSpherA2, an implementation of non-spherical atom-form-factors.[9] NoSpherA2 implementation of HAR makes use of tailor-made aspherical atomic form factors calculated from a Hirshfeld-partitioned electron density (ED) not from spherical-atom form factors. The ED was calculated from a Gaussian basis set single determinant SCF wavefunction from DFT using selected functionals for a fragment of this crystal. The following options were used: SOFTWARE: ORCA PARTITIONING: NoSpherA2 INT ACCURACY: Normal METHOD: PBE BASIS SET: def2-TZVP CHARGE: 0 MULTIPLICITY: 1 DATE: 2022-01-13_17-40-03

There is a single molecule in the asymmetric unit, which is represented by the reported sum formula. In other words: Z is 4 and Z' is 1.

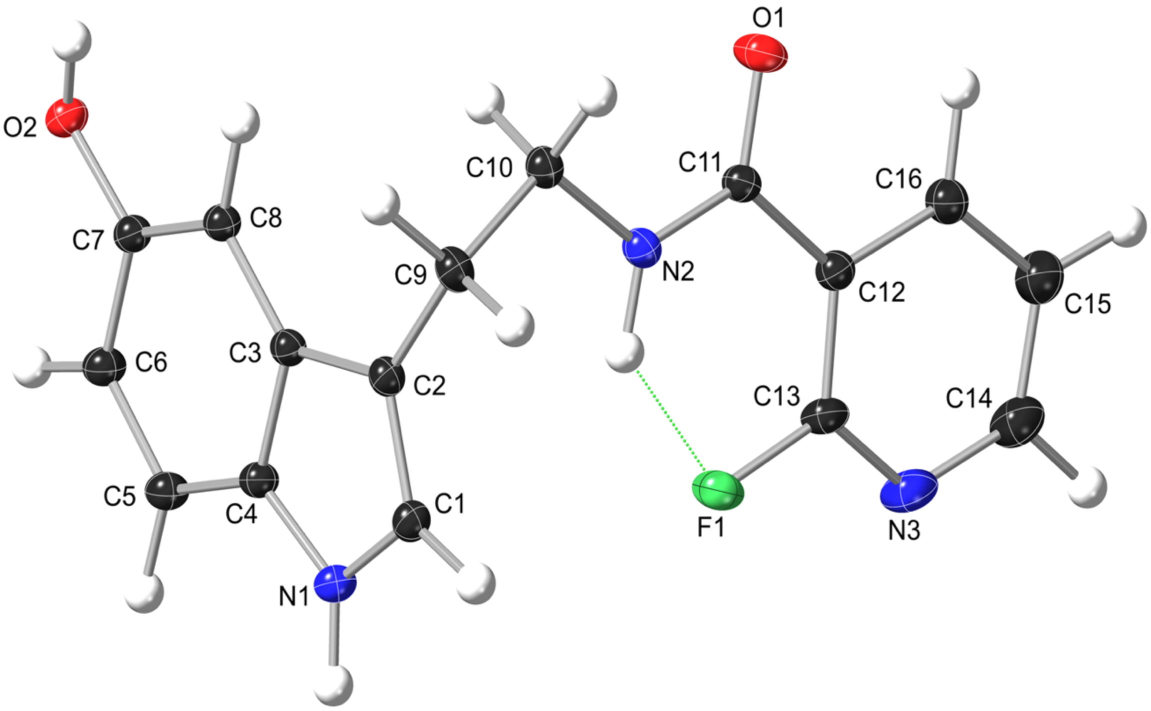

S5 Fig: Thermal ellipsoid representation of HIFN (**5**).

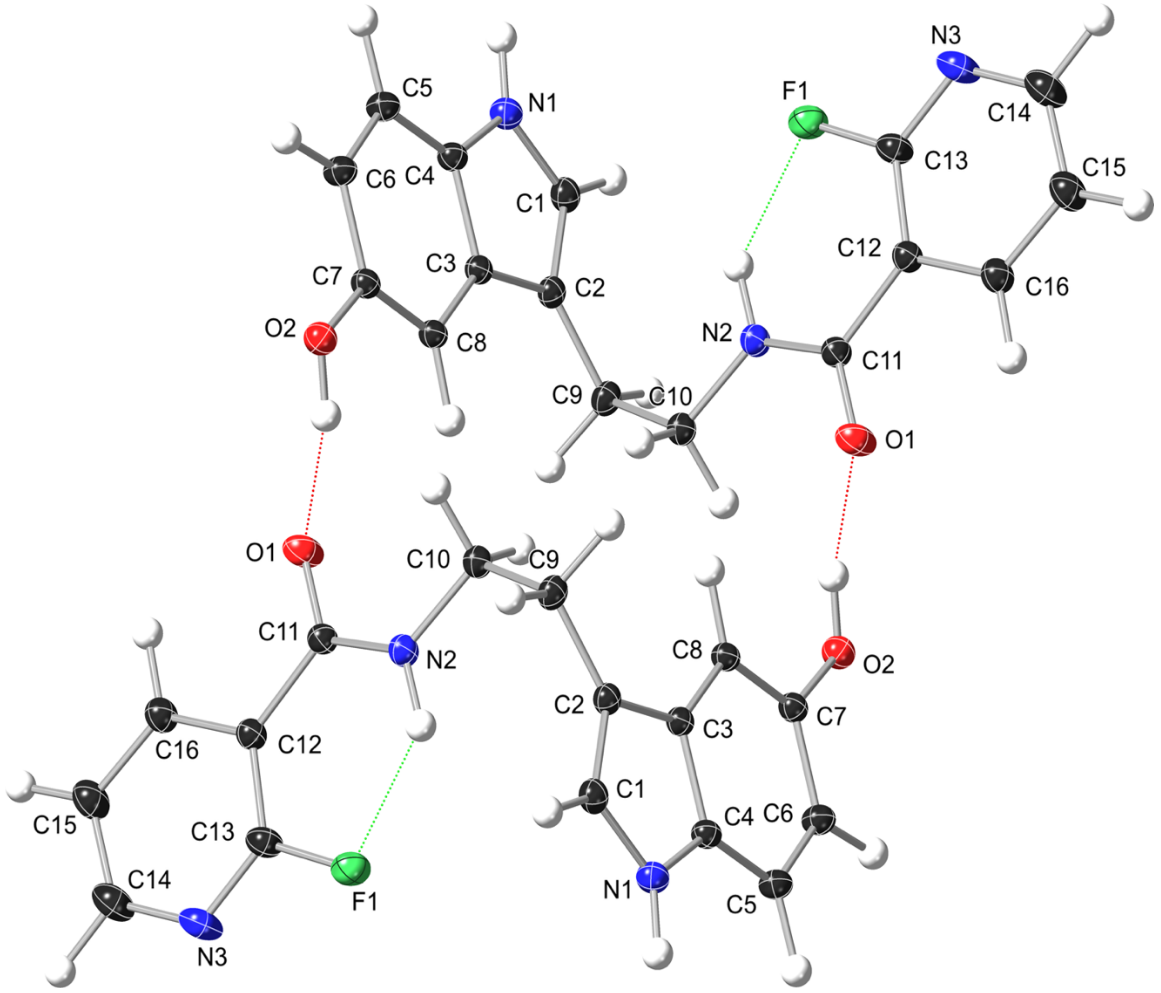

S6 Fig: Hydrogen bonded dimer in the crystal structure of HIFN (**5**).

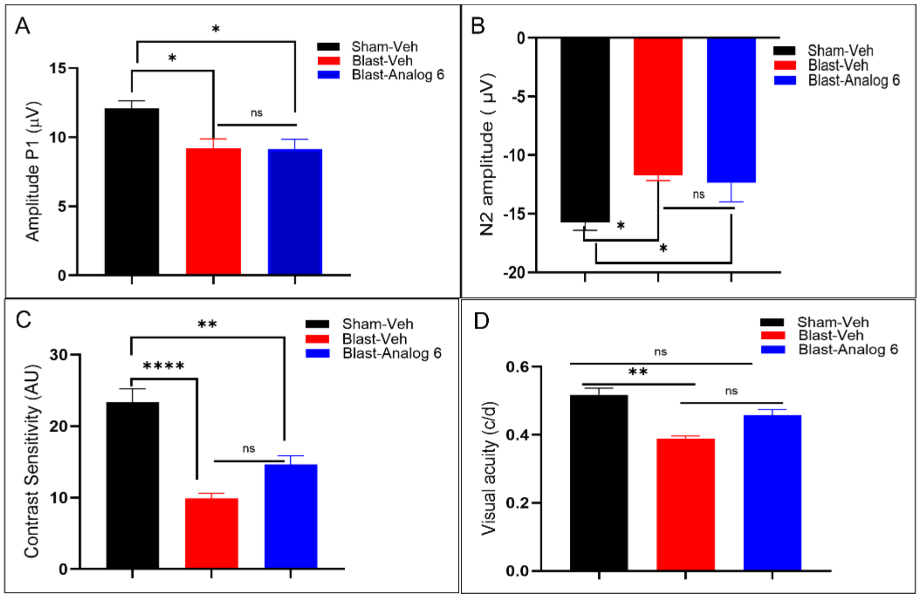

S7 Fig. Analog 6 did not exhibit in-vivo neuroprotection. Animals were treated with analog **6** (ip, 40mg/kg) following blast (same regime was followed as for HIFN (**5**) treatment shown in Figure 2A). No statistically significant change in P1 (A) and N2 (B) wave was seen in animals treated with analog **6** compared to vehicle treated animals. Analog **6** treatment did not rescue the visual function deficit; no statistically significant improvement in contrast sensitivity (C) and visual acuity (D) was observed in treated animals. * p≤0.05; **p≤0.01 ;****p≤0.0001, n=4-5/group.

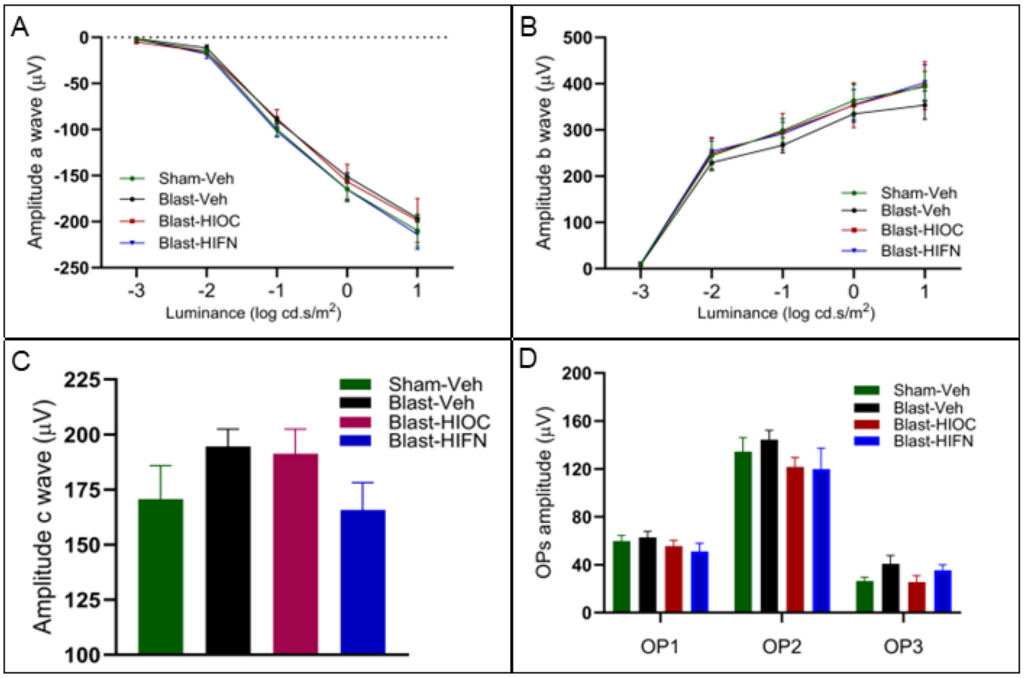

S8 Fig. Outer retinal neurons were not affected by overpressure blast injury. At 9 weeks post blast, animals did not show any change in rod photoreceptor or rod bipolar cell

function as assessed by ERG: (A) amplitude of ‘a-wave’; (B) amplitude of ‘b-wave’. Function of RPE and amacrine cells was also not altered as shown by: (C) ‘c-wave’ and (D) oscillatory potentials (OP), respectively**.** No significant differences were found for any of the parameters recorded. Data are expressed as mean ± SEM; n=6-8/group.

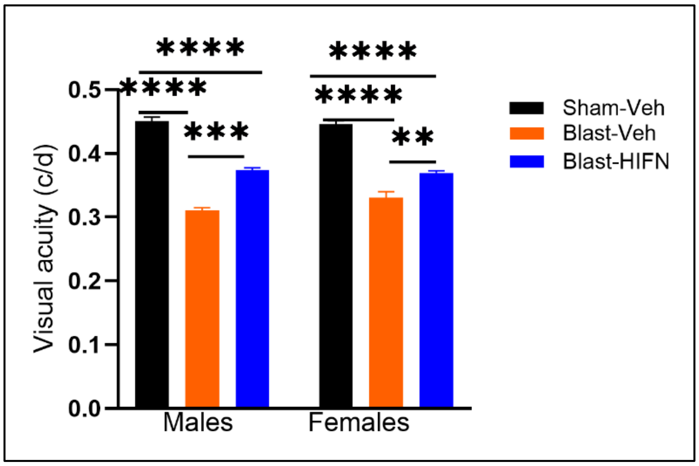

S9 Fig. HIFN reduces visual function decline in females and males. Visual acuity was tested in male and female mice to screen sex-specific effects . Mice were exposed to blast and treated with HIFN (40 mg/kg) or vehicle 30 minutes later and daily for the next 6 days. Visual acuity was measured 8 days after blast exposure. **$p \le 0.01$; ***$p \le 0.001$; ****$p \le 0.0001$, n=5-6/group.

S2 Table. Electroretinogram 'a-wave' and 'b-wave' recordings 3 and 6-week post blast showing no significant effect on rod photoreceptor and bipolar cell function.

a-wave amplitude (3week post blast)					
Luminance	Sham-Veh	Blast-Veh	Blast-HIOC (**1**)	Blast-HIFN (**5**)	p-value
0.001 cd.s/m^2	-3.44±1.25	-2.96±1.61	-2.84±0.76	-2.35±0.46	0.993
0.005 cd.s/m^2	-7.44±1.74	-10.69±1.71	-8.84±1.49	-7.05±1.79	0.463
0.01 cd.s/m^2	-20.42±2.34	-21.39±1.91	-24.15±2.54	-19.24±2.20	0.505
0.1 cd.s/m^2	-108.53±5.42	-105.34±6.70	-118.05±9.54	-109.17±6.07	0.628
1 cd.s/m^2	-189.27±10.04	-185.21±9.56	-202.66±12.41	-200.78±10.44	0.589
b-wave amplitude (3week post blast)					
0.001 cd.s/m^2	10.13±1.16	7.83±1.62	9.00±1.08	8.54±1.49	0.725
0.005 cd.s/m^2	258.94±19.07	236±20.68	263.10±16.29	277.23±16.6	0.483
0.01 cd.s/m^2	302.07±19.99	272.05±21.72	292.65±18.07	316.40±15.02	0.438
0.1 cd.s/m^2	351.55±20.59	308.65±21.46	329.66±19.10	373.30±12.93	0.131
1 cd.s/m^2	425.18±25.77	398.94±28.39	399.60±24.68	453.45±22.87	0.434
a-wave amplitude (6week post blast)					
0.001 cd.s/m^2	-3.63±0.75	-3.32±1.66	3.81±1.13	-2.81±1.75	0.962
0.005 cd.s/m^2	-11.64±3.05	14.38±2.86	-8.82±1.92	-7.37±1.80	0.251
0.01 cd.s/m^2	-22.29±3.06	-23.41±2.84	-13.79±2.28	-12.24±2.77	0.01
0.1 cd.s/m^2	-92.32±5.99	-104.60±4.73	-102.13±9.22	-92.98±11.90	0.608
1 cd.s/m^2	-158.15±12.17	-167.78±6.45	-167.76±11.54	-159.71±16.22	0.900
b-wave amplitude (6week post blast)					
0.001 cd.s/m^2	7.65±0.49	8.72±1.53	11.28±1.57	10.75±1.77	0.243
0.005 cd.s/m^2	216.75±15.25	209.05±19.44	226.20±23.51	217.85±27.93	0.954
0.01 cd.s/m^2	243.72±15.84	244.22±17.19	260.01±23.58	242.53±27.19	0.928
0.1 cd.s/m^2	288.15±25.34	293.41±14.36	301.10±22.57	293.41±27.26	0.982
1 cd.s/m^2	353.62±31.55	350.31±18.15	356.11±25.08	344.98±30.57	0.992

SI References

1. Setterholm NA, McDonald FE, Boatright JH, Iuvone PM. Gram-scale, chemoselective synthesis of N-[2-(5-hydroxy-1H-indol-3-yl) ethyl]-2-oxopiperidine-3-carboxamide (HIOC). Tetrahedron Letters. 2015 Jun 3;56(23):3413-5.
2. Weingarten MD, Prein M, Price AT, Snyder JP, Padwa A. Theoretical Insights Regarding the Cycloaddition Behavior of Push− Pull Stabilized Carbonyl Ylides. The Journal of Organic Chemistry. 1997 Apr 4;62(7):2001-10.
3. Yamada K, Teranishi S, Miyashita A, Ishikura M, Somei M. A novel synthesis of 3,4,5,6-tetrahydro-7-hydroxy-1H-azepino[5,4,3-*cd*]indole derivatives from serotonin. Heterocycles. 2011 Oct; 83(11):2547-62. (Doctoral dissertation, Kanazawa University).
4. Walsh MP, Barclay JA, Begg CS, Xuan J, Kitching MO. Conglomerate crystallization in the cambridge structural database (2020–2021). Crystal Growth & Design. 2023 Mar 22;23(4):2837-44.
5. Sheldrick GM. *SHELXT* - Integrated space-group and crystal-structure determination. Acta Crystallographica. 2015 Jan;A71(1):3-8.
6. Dolomanov OV, Bourhis LJ, Gildea RJ, Howard JAK, Puschmann H. *OLEX*2: a complete structure solution, refinement and analysis program. Journal of Applied Crystallography. 2009 Apr;42(42):339-41.
7. Bourhis LJ, Dolomanov OV, Gildea RJ, Howard JAK, Puschmann H. The anatomy of a comprehensive constrained, restrained refinement program for the modern computing environment - *Olex2* dissected. Acta Crystallographica. 2015 Jan;A71(1):59-75.
8. Sheldrick GM. Using phases to determine the space group. Acta Crystallographica. 2018;a353.
9. Kleemiss F, Dolomanov OV, Bodensteiner M, Peyerimhoff N, Midgley L, Bourhis LJ, Genoni A, Malaspina LA, Jayatilaka D, Spencer JL, White F, Grundkötter-Stock B, Steinhauer S, Lentz D, Puschmann H, Grabowsky S. Accurate crystal structures and chemical properties from NoSpherA2. Chemical Science. 2021;12:1675-92.
10. Min J, Wang C, Wang L. A new method for detecting intramolecular H-bonds of aromatic amides based on the de-shielding effect of carbonyl groups on β-protons. Physical Chemistry Chemical Physics. 2021;23(23):13284-91.
